# Metabolomics-Based Analysis Reveals Differences in Non-Volatile Components of *Artemisiae argyi* Folium with Different Aging Durations

**DOI:** 10.3390/molecules31213995

**Published:** 2026-11-05

**Authors:** Jiaojiao Cai, Hanxiang Li, Chuang Liu, Yanbin Liu, Qiaoli Wang, Xiaofeng Liao, Sichen Mao, Yaguan Zeng, Wei Zhang

**Affiliations:** 1 School of Traditional Chinese Medicine, Guangdong Pharmaceutical University, Guangzhou 510006, China; caijiaojiao@scbg.ac.cn; 2 Guangdong Key Laboratory of Applied Botany, South China Botanical Garden, Chinese Academy of Sciences, Guangzhou 510650, China; maosichen@scbg.ac.cn (S.M.); zengyaguan24@mails.ucas.ac.cn (Y.Z.); 3 South China National Botanical Garden, Guangzhou 510650, China; lihanxiang@scbg.ac.cn; 4 Tea-Mugwort Industry Development Center of Tongbai County, Nanyang 474750, China; lc82002299@163.com (C.L.); liuyanbin@scbg.ac.cn (Y.L.); 5 *Artemisia argyi* Branch Center, National Engineering Research Center for Modernization of Traditional Chinese Medicine, Guangzhou 510610, China; wangql@gzjnsw.com; 6 College of Life Science and Technology, Jinan University, Guangzhou 510610, China; liaoxf@gzjnsw.com; 7 University of Chinese Academy of Sciences, Beijing 100049, China

**Keywords:** *Artemisiae argyi* Folium, untargeted metabolomics, non-volatile metabolites, candidate markers, aging duration, UPLC-Orbitrap-MS/MS

## Abstract

*Artemisiae argyi* Folium (AAF) is traditionally recognized to exhibit improved quality with prolonged aging duration. However, the differences in its non-volatile metabolite profile among AAF samples with different aging durations are still poorly characterized. In this study, ultra-performance liquid chromatography coupled to Orbitrap tandem mass spectrometry (UPLC-Orbitrap-MS/MS) in positive ion mode, combined with metabolomics approaches, was used to analyze AAF samples representing different aging durations from 0 to 7 years. A total of 138 compounds were putatively annotated across the eight AAF groups. Principal component analysis (PCA) and partial least squares discriminant analysis (PLS-DA) showed distinct metabolic separation among the eight sample groups. Statistical analysis further identified 48 differential metabolites that satisfied the screening criteria. Mfuzz clustering classified the 48 differential metabolites into three major temporal patterns: an overall decline, higher relative abundances in the middle-to-late and early aging-duration groups. Furthermore, nine exploratory candidate markers associated with aging duration were selected, including kaempferol, apigenin, luteolin, rutin, esculetin, isofraxidin, ferulic acid, argyinolide H, and matricarin. Collectively, these results show new insights into the metabolomic differences associated with different aging durations and lay the foundation for screening aging-duration-associated candidate markers and future quality evaluation of AAF.

## 1. Introduction

*Artemisiae argyi* Folium is the dried leaf of *Artemisia argyi* Lévl. et Vant., characterized by a diverse chemical composition, broad pharmacological activities, and wide-ranging applications. According to the Chinese Pharmacopoeia (2025) [1], AAF is warm in nature and pungent and bitter in flavor and is traditionally associated with the liver, spleen, and kidney meridians. Its traditional therapeutic effects include warming meridians to arrest bleeding, dispelling cold, and alleviating pain. Modern phytochemical studies have demonstrated that AAF contains a diverse array of chemical compounds, predominantly essential oils, flavonoids, phenolic acids, coumarins, terpenoids, and polysaccharides. These compounds possess a wide range of biological activities, such as antioxidant, anti-inflammatory, antibacterial, analgesic, and immunoregulatory activities [2,3]. Beyond its established conventional medicinal value, AAF has been extensively developed and applied in healthcare products, functional foods, and cosmetics in recent years [4]. Driven by increasing market demand, large-scale AAF cultivation has expanded rapidly, leading to a continuous increase in total yield. In 2021, the AAF cultivation area in China exceeded 40,000 hectares, or approximately 600,000 mu; by 2024, the total cultivation area of fresh yield of AAF (newly harvested AAF) was over 50,000 hm^2^ [5,6]. Nevertheless, commercially available AAF products may vary in quality. The quality variation of AAF is derived from multiple sources, including geographical origin, harvest time, post-harvest processing techniques, and storage conditions [7]. Among these factors, aging duration has been recognized as an important characteristic associated with variations in the chemical composition and quality of AAF.

Much of the research on variations in the chemical composition and quality assessment of *Artemisiae argyi* Folium (AAF) has focused on volatile compounds. Studies using gas chromatography–mass spectrometry (GC–MS) and the electronic nose have elucidated the differences in essential oil composition among different germplasm resources. Previous studies have focused on the volatile constituents of AAF aged for 1, 3, and 5 years and have identified candidate markers, while recent studies on aging have primarily examined the patterns of essential oil changes over relatively short periods (ranging from 0–150 days to 1–2 years) [8,9,10,11]. In contrast, research into non-volatile constituents has largely been confined to total flavonoid or polysaccharide content, chromatographic fingerprint profiles, or a small number of target compound groups [12,13,14]; there remains a lack of sufficient understanding of non-volatile metabolomic profiles analyzed by year. One study used UPLC–Orbitrap–MS/MS combined with GC–MS to analyze AAF samples aged for 1, 3, and 5 years, identifying seven flavonoids and nine terpenoids as candidate markers associated with aging [9]. However, this study focused on three non-consecutive aging durations (1, 3, and 5 years), leaving metabolic differences among samples representing consecutive aging durations insufficiently characterized. During long-term aging, non-volatile metabolites of AAF, particularly flavonoids, phenolic acids, terpenoids, and related glycosides, commonly undergo chemical transformations including hydrolysis, oxidation, and isomerization, which substantially reshape its metabolic profile [13,15,16]. In addition, aging processing has been proven to enhance the in vitro antioxidant capacity of AAF polysaccharides against hydroxyl radicals, superoxide anions, ABTS, and DPPH radicals. Notably, polysaccharides from 5-year-aged AAF exhibited significantly superior antioxidant activity compared with those from 1- and 3-year-aged samples, while 3-year-aged AAF polysaccharides possessed the optimal immunomodulatory activity [17]. Despite these advances, most previous studies have mainly focused on essential oils, total active contents, chromatographic fingerprints, or a limited number of target compounds. Differences in non-volatile metabolomic profiles among AAF samples representing consecutive aging durations remain insufficiently characterized.

Therefore, there is a need for analytical methods to characterize AAF metabolites and compare their changes during aging. Mass spectrometry-based metabolomics has emerged as a powerful and high-throughput analytical tool for profiling a broad range of small molecules in complex plant and biological systems [18]. This technology has been widely applied in traditional Chinese medicine research, including chemical composition characterization, geographical origin discrimination, processing efficacy evaluation, and exploration of metabolic variations caused by temporal and environmental factors [19,20,21,22]. Furthermore, ultra-performance liquid chromatography coupled to Orbitrap tandem mass spectrometry (UPLC-Orbitrap-MS/MS) offers advantages such as high sensitivity and high resolution, enabling the rapid analysis of structurally diverse non-volatile constituents in complex traditional Chinese medicines; this method has been successfully applied to a variety of medicinal plants, including *Artemisiae argyi* Folium, *Atractylodis macrocephalae* Rhizoma, *Zingiberis* Rhizoma, and *Hibisci mutabilis* Folium [9,23,24,25]. Compared with traditional analytical methods that only detect total component contents or limited targeted markers, untargeted metabolomics combined with UPLC-Orbitrap-MS/MS enables simultaneous profiling of hundreds of metabolites and identification of characteristic compounds with unique temporal variation trends [18,26], making it suitable for comparative characterization of AAF metabolomic profiles across different aging durations.

In this study, a metabolomic approach based on UPLC-Orbitrap-MS/MS in positive ion mode was applied to profile the non-volatile metabolites of freshly harvested AAF samples and of AAF samples aged for 1 to 7 years. Our study aimed to address the following questions: (1) whether non-volatile metabolites of AAF exhibit significant changes with different aging durations, and (2) whether candidate metabolite markers can be identified for discriminating AAF with different aging durations. The findings of this work would help characterize metabolomic variation among AAF samples representing different aging durations and provide a metabolomics-based basis for evaluating chemical differences among AAF samples with different aging durations.

## 2. Results

### 2.1. Non-Volatile Metabolite Profiling and Putative Annotation

**Figure 1:**
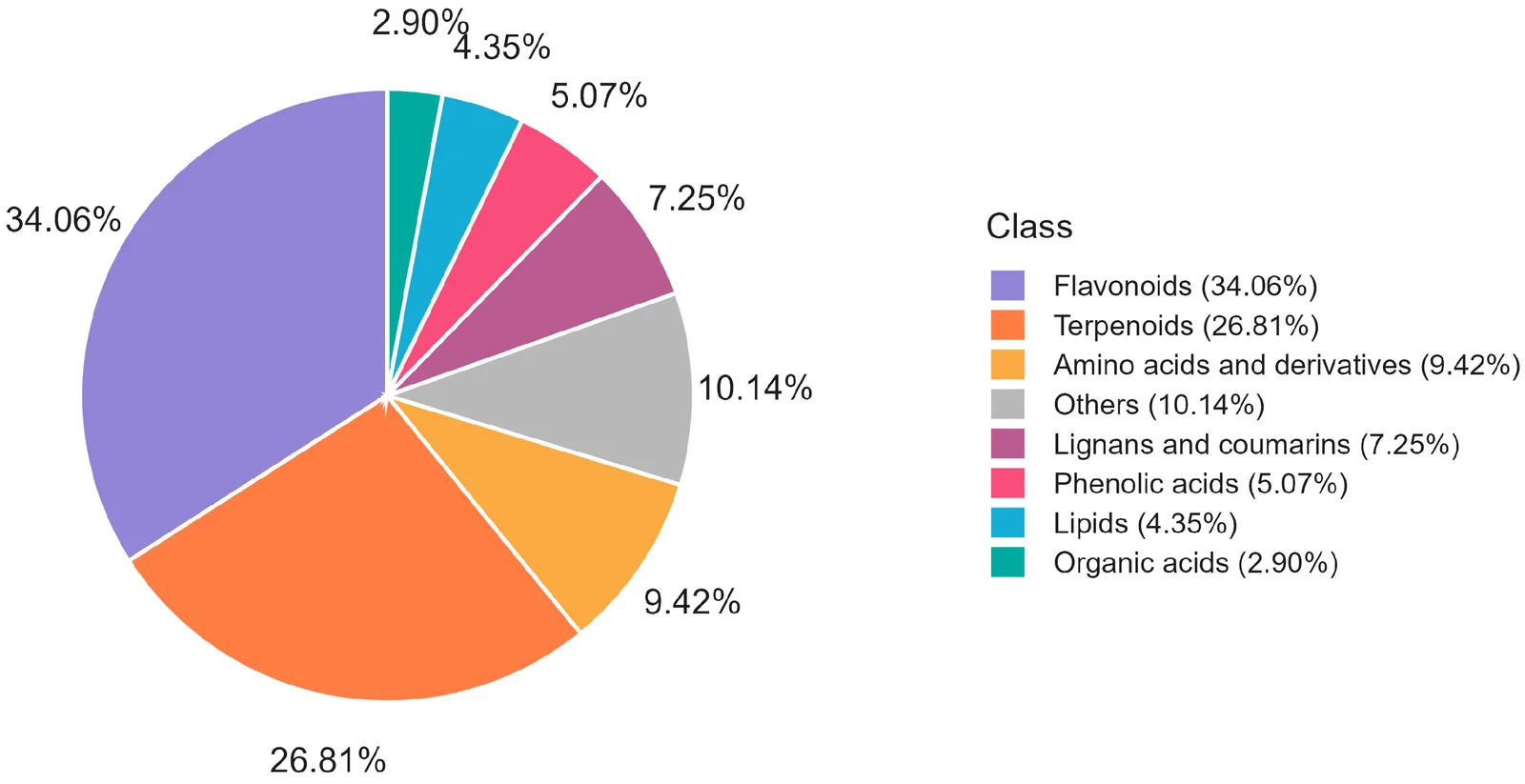
Distribution of 138 putatively annotated non-volatile metabolites by chemical class.

Untargeted metabolomic profiling based on UPLC-Orbitrap-MS/MS in positive ion mode was performed to characterize the non-volatile chemical metabolites of freshly harvested AAF (FA) and AAF samples aged for 1–7 years (designated AAF-1y, AAF-2y, AAF-3y, AAF-4y, AAF-5y, AAF-6y, and AAF-7y, respectively). The corresponding total ion chromatograms (TICs) of all samples are shown in Figure S1. Based on accurate mass measurements, chromatographic retention behaviors, MS/MS fragmentation patterns, database matching, and previously reported phytochemical evidence, a total of 138 non-volatile metabolites were putatively annotated in positive-ion mode (Table S1). All annotated compounds were categorized into eight major classes, including 47 flavonoids, 37 terpenoids, 13 amino acids and derivatives, 10 lignans and coumarins, 7 phenolic acids, 6 lipids, 4 organic acids, and 14 others (Figure 1, Table S1).

### 2.2. Multivariate Analysis of AAF Samples Across Aging Durations

**Figure 2:**
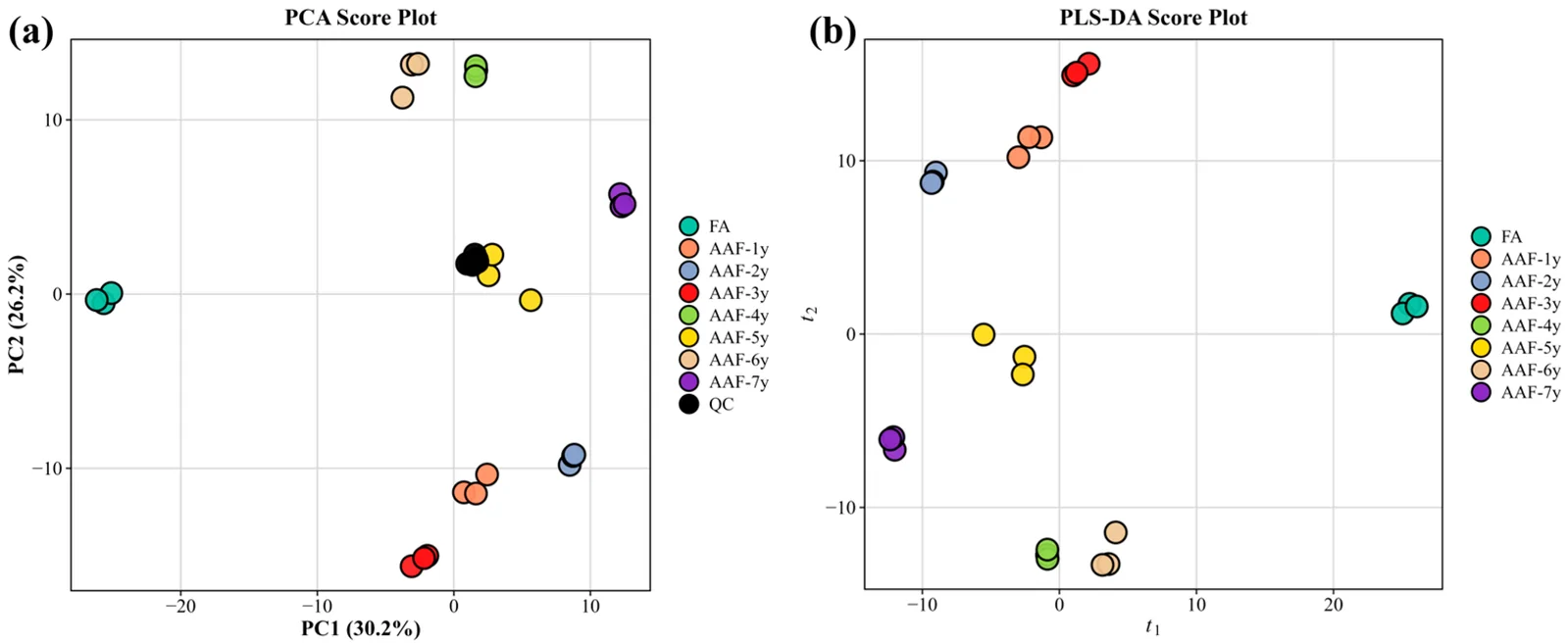
Multivariate score plots of non-volatile metabolic profiles in *Artemisiae argyi* Folium (AAF). (**a**) Principal component analysis (PCA). (**b**) Partial least-squares discriminant analysis (PLS-DA).

Principal component analysis (PCA) and partial least squares discriminant analysis (PLS-DA) were used to characterize overall metabolic differences among samples and assess separation among aging-duration groups. PCA was performed to visualize intergroup metabolic disparities and intragroup variability across all AAF samples. PC1 and PC2 explained 30.2% and 26.2% of the total variance, respectively, with a cumulative explained variance of 56.4% (Figure 2a). The FA group was distinctly separated from all aged samples, while samples aged for 1–3 years exhibited highly similar metabolic profiles and clustered closely. In comparison, AAF samples aged for 4–7 years formed an independent cluster, with a pronounced separation in PCA space between the 3-year and 4-year groups. The close clustering of quality control (QC) samples indicates that this analytical platform maintained good stability throughout the analytical sequence.

Supervised PLS-DA was further used to maximize intergroup metabolic differentiation in the eight AAF sample groups. The four-component PLS-DA model showed goodness-of-fit and predictive performance (Figure 2b), with cumulative parameters of R^2^X = 0.80, R^2^Y = 0.57, and Q^2^ = 0.56. A 1000-permutation test supported the statistical validity of the PLS-DA model, as the original R^2^Y and Q^2^ values were higher than those of randomly permuted models (*p* = 0.001 for each statistic). Permutational multivariate analysis of variance (PERMANOVA) detected significant differences in overall metabolic profiles among the aging-duration groups (F = 30.61, R^2^ = 0.93, *p* = 0.0001) (Figure S13). In addition, the test for homogeneity of multivariate dispersions (PERMDISP) showed no significant difference (F = 1.86, *p* = 0.14) (Figure S14). Furthermore, PLS regression analysis was performed by treating aging duration as a continuous variable to explore its correlation with metabolic profiles. The model exhibited excellent fitting and predictive performance, with cumulative R^2^X, R^2^Y, and Q^2^ values of 0.77, 0.96, and 0.92, respectively (Figure S15). The corresponding permutation test yielded significant results (*p* = 0.001 for each statistic), suggesting an association between the non-volatile metabolic profiles and aging duration in the investigated AAF samples.

### 2.3. Screening and Analysis of Candidate Differential Metabolites

**Figure 3:**
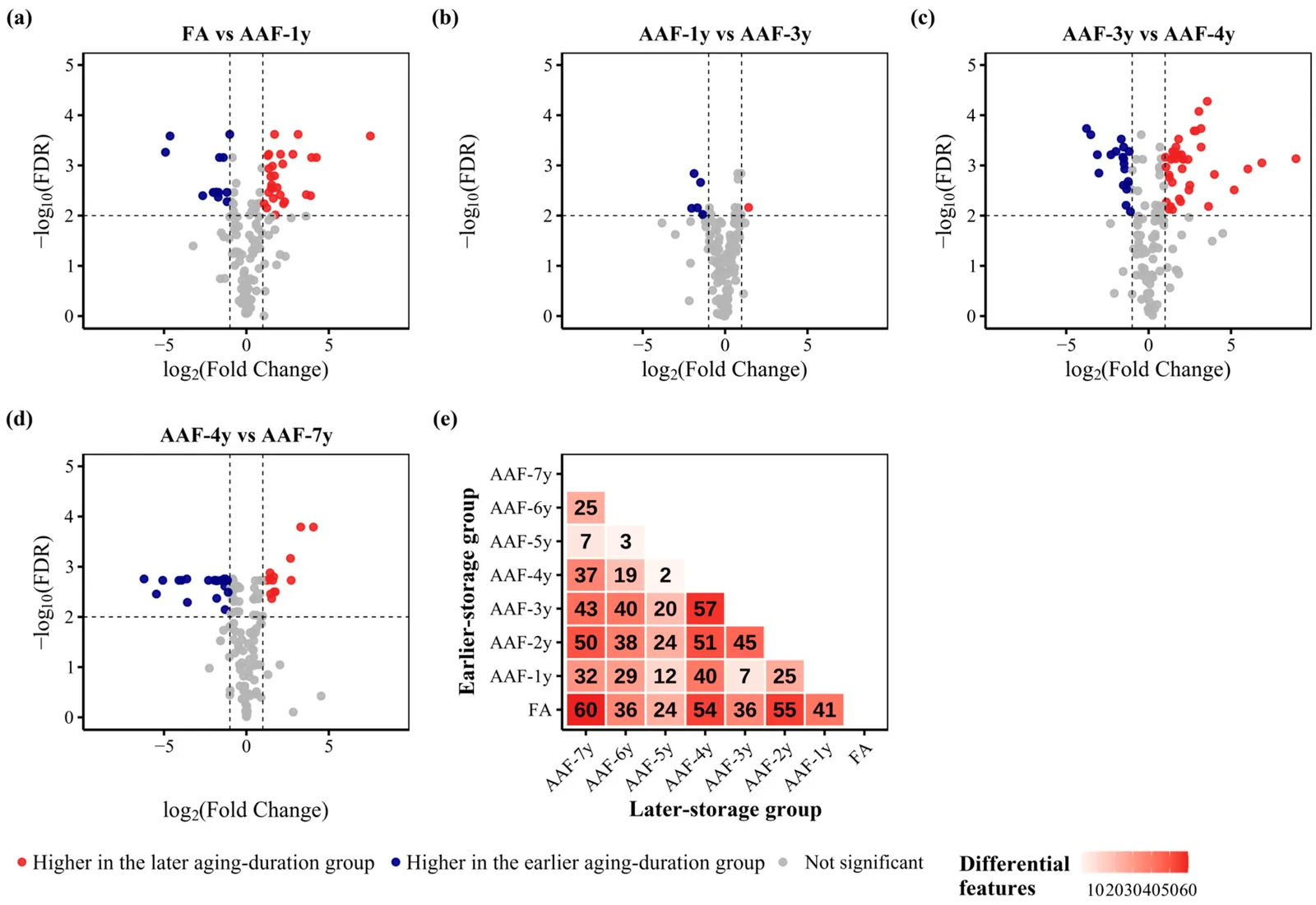
Pairwise differential metabolite analysis of AAF samples representing different aging durations. (**a**–**d**) Volcano plots showing differential metabolites between FA and AAF-1y, AAF-1y and AAF-3y, AAF-3y and AAF-4y, and AAF-4y and AAF-7y. (**e**) Matrix of differential-metabolite counts for all 28 pairwise contrasts. Accordingly, (**a**–**d**) are representative volcano plots used to visually illustrate the differences in metabolite distribution across different aging-duration groups; (**e**) shows the main results of the comparative analysis.

PCA and PLS-DA both revealed clear differences in the overall metabolic profiles of AAF samples across eight aging durations. To identify key metabolites contributing to metabolic differences among different aging-duration groups, pairwise differential metabolite screening was performed using the combined criteria of |log_2_ FC| > 1 and FDR < 0.01. To characterize differences among AAF aging-duration groups, volcano plots were generated based on intergroup comparisons representing the early, mid, and late stages of aging (Figure 3a–d); a total of 41 differential metabolites were detected between FA and AAF-1y, indicating a marked difference between these groups. In contrast, only 7 differential metabolites were detected between AAF-1y and AAF-3y, suggesting that overall metabolic differences were relatively limited during this period. Notably, the number of differential metabolites between AAF-3y and AAF-4y increased markedly to 57, after which it declined overall, indicating a pronounced difference in metabolic profiles between the AAF-3y and AAF-4y groups. A total of 37 differential metabolites were also detected between AAF-4y and AAF-7y, indicating differences between the long-aged groups. Further pairwise comparisons among aging-duration groups revealed marked differences in the numbers of differential metabolites (Figure 3e). As shown in the figure, the number of differential metabolites in AAF does not follow a monotonic trend with increasing aging duration; instead, it fluctuates by time stage, as shown in the corresponding volcano plots (Figures S4–S10).

**Figure 4:**
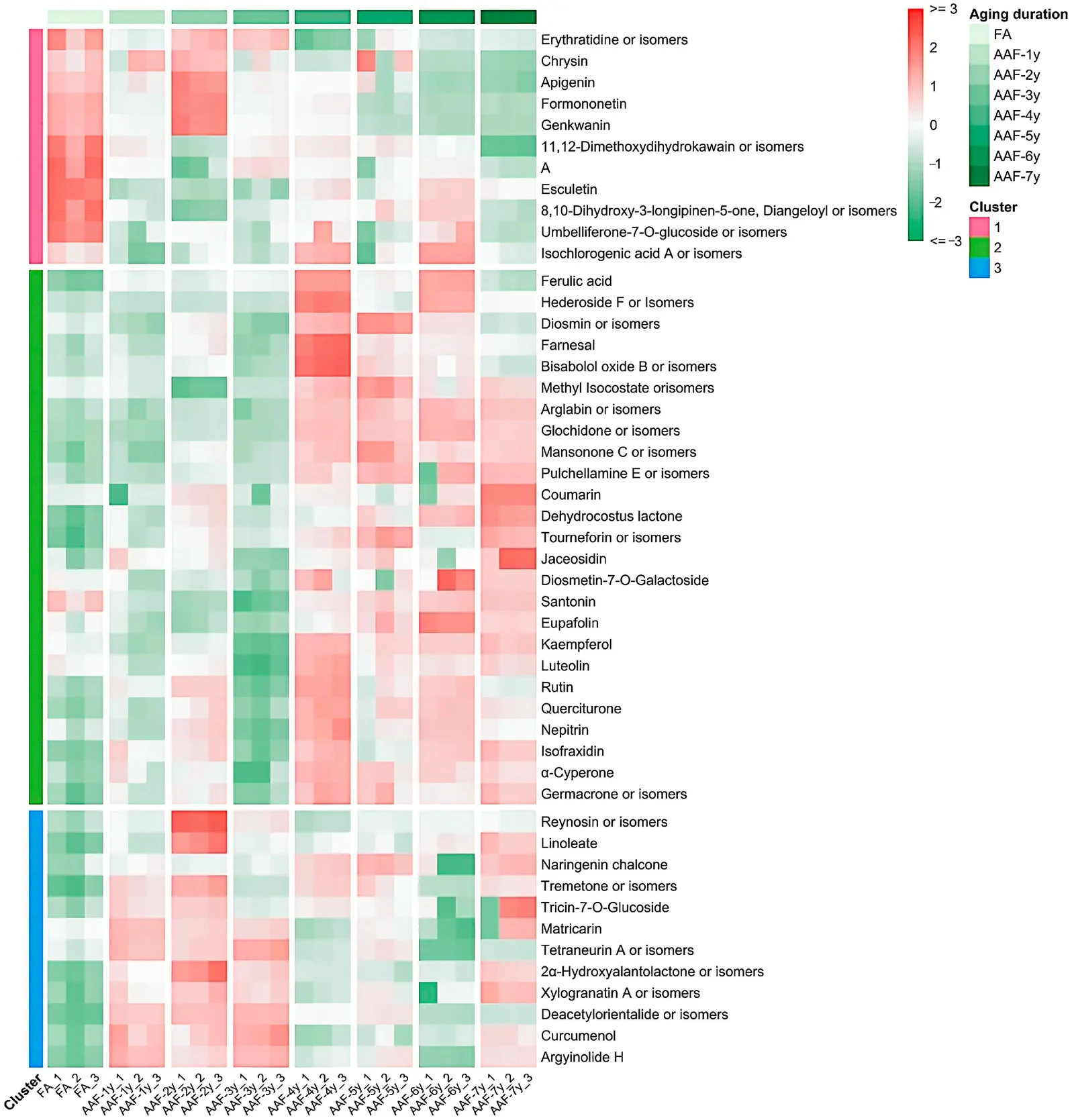
Heatmap of 48 differential metabolites grouped by Mfuzz cluster. Columns represent biological samples (*n* = 3 per group), and colors indicate row-wise Z-scores of log_2_-transformed relative abundances. Among them, the differential metabolite A represents [(3aR,4S,6Z,9Z,11aS)-4-hydroxy-6,10-dimethyl-3-methylidene-2,8-dioxo-4,5,11,11a-tetrahydro-3aH-cyclodeca[b]furan-11-yl](Z)-2-methylbut-2-enoate or its isomers.

Metabolites were selected based on the PLS-DA models, with VIP ≥ 1 as the criterion. Among the 28 pairwise comparisons, metabolites for which at least one pair met the criteria of |log_2_ FC| > 1 and FDR < 0.01 were retained. After removing duplicated metabolites across comparisons, 48 differential metabolites were ultimately identified. The annotated metabolites were primarily classified into flavonoids, terpenoids, lignans and coumarins, phenolic acids, and other metabolites. Detailed annotation information and a summary of the differences are presented in Table S1. Among all 28 comparisons, 48 metabolites with significant differences are indicated by an asterisk (*). A heatmap was generated to visualize the relative abundance patterns of the 48 differential metabolites across all 24 biological samples (Figure 4). Metabolic profiles within each aging period were highly consistent across the three biological replicates. In contrast, obvious metabolic differences were detected among samples with different aging durations, indicating distinct non-volatile metabolic characteristics among AAF samples with different aging durations. The color gradient in the heatmap represents the normalized relative abundance of individual metabolites, with red and green indicating relatively high and low abundance, respectively.

**Figure 5:**
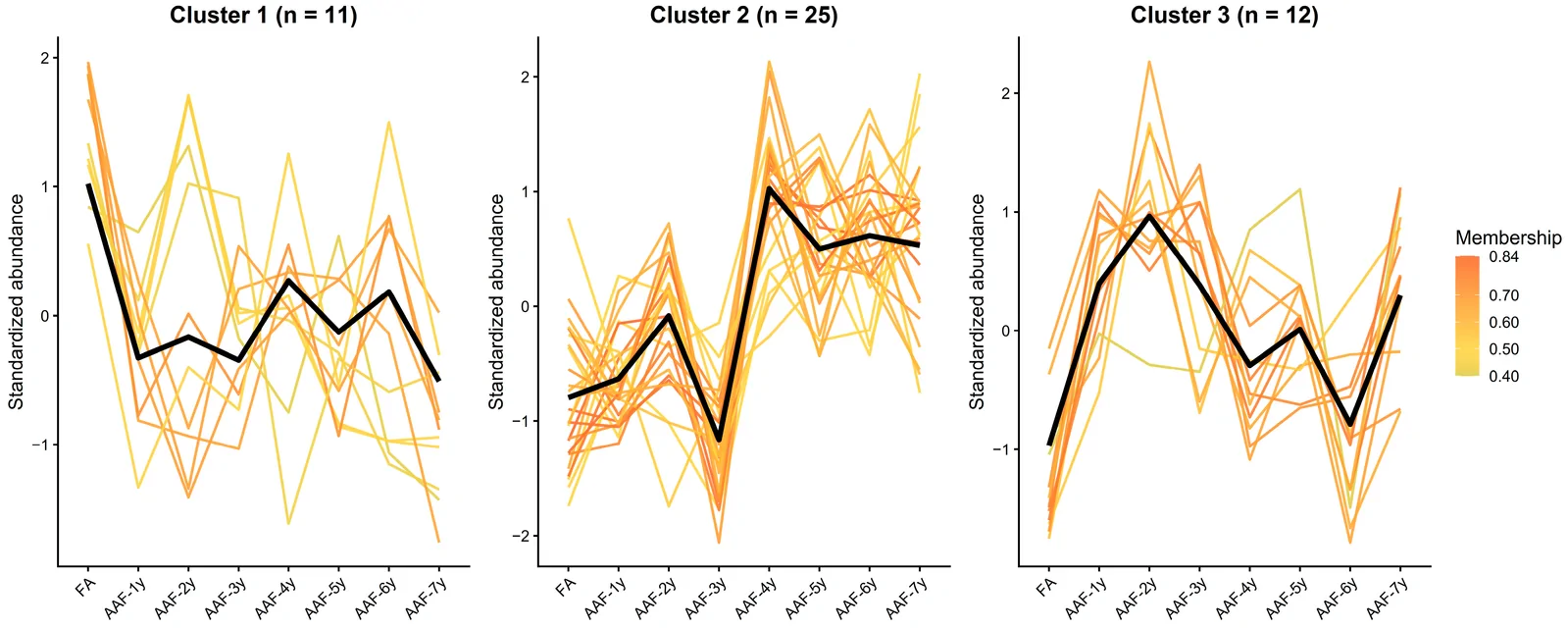
Mfuzz clustering of abundance patterns of 48 differential metabolites across different aging durations of AAF. Thin lines of different colors represent the temporal abundance profiles of individual metabolites; the color of each line indicates its Mfuzz membership value, with warmer colors indicating a stronger association between the metabolite and its cluster pattern. The thick black line represents the trajectory of the cluster centroid profile. The number of metabolites in each cluster is indicated in parentheses.

Based on the group-mean relative-abundance profiles, Mfuzz clustering identified three characteristic abundance patterns among the 48 preselected differential metabolites (Figure 5). These patterns summarize the variations among different aging-duration groups, but they cannot serve as independent evidence for the existence of distinct aging stages. Cluster 1 comprised 11 metabolites whose normalized relative abundances were generally higher in fresh samples (FA), decreased markedly after aging, and remained relatively low during subsequent aging phases. The relative abundances of some differential compounds showed a significant increase in AAF-2y, such as apigenin, formononetin, and genkwanin. Cluster 2 comprised 25 metabolites, representing the largest group. Their relative abundances were generally low from the FA stage through the AAF-3y stage, but rose markedly at the AAF-4y stage and remained generally high through the AAF-7y stage, showing higher relative abundances in the AAF-4y to AAF-7y groups. This cluster included a large number of flavonoids commonly found in AAF, such as kaempferol, luteolin, rutin, and jaceosidin. Cluster 3 comprised 12 metabolites that were generally low in FA, increased during 1–3 years of aging, decreased from AAF-4y to AAF-6y, and rose again in AAF-7y. This cluster included the terpenoid compounds argyinolide H and matricarin, which have been reported in AAF.

Collectively, these clustering results revealed aging-duration-associated and metabolite-specific relative-abundance patterns. Different metabolites exhibited distinct relative-abundance patterns across the aging-duration groups, including an overall decline, mid-to-late-stage enrichment, and early-stage enrichment with subsequent fluctuations. Therefore, the data from this study did not show a uniform monotonic pattern in relative abundances across the investigated groups.

### 2.4. Relative Abundance of Exploratory Candidate Differential Markers

**Figure 6:**
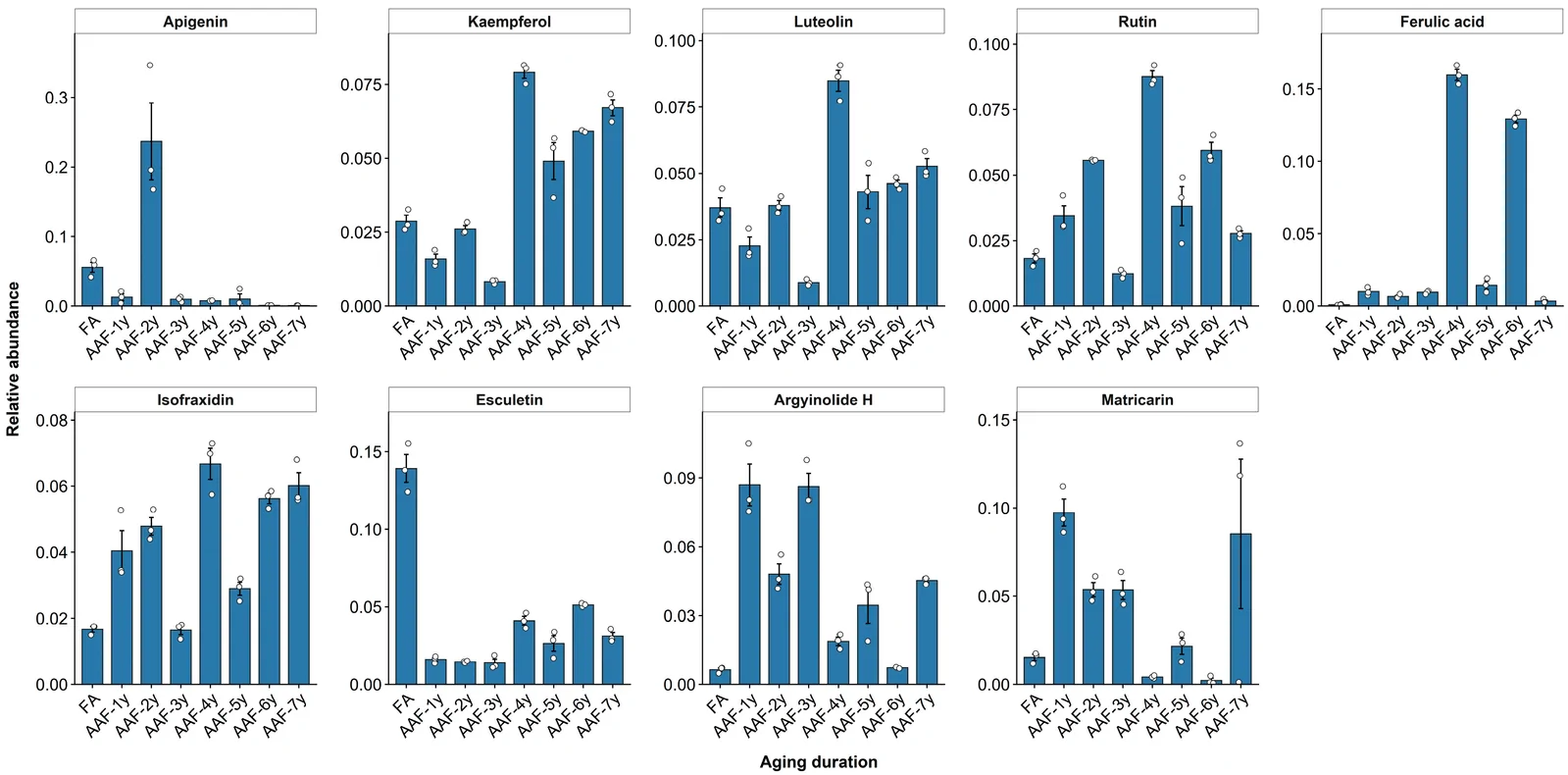
Relative abundance of nine exploratory candidate markers across nominal AAF aging-duration groups. Bars show group means with standard errors; points show individual values.

Nine representative compounds were selected qualitatively from the 48 differential metabolites based on their previously reported occurrence in AAF, their representation of different chemical classes, and their coverage of the three observed relative abundance patterns (Figure 6). These compounds were considered representative exploratory candidate markers for further investigation. These selected compounds comprised four flavonoids (kaempferol, apigenin, luteolin, and rutin), two coumarins (esculetin and isofraxidin), one phenolic acid (ferulic acid), and two terpenoids (argyinolide H and matricarin) (Table 1). The nine candidate aging markers exhibited distinct temporal patterns. Esculetin and apigenin were relatively abundant in FA and generally decreased with increasing aging duration. However, esculetin showed some recovery at AAF-4y and AAF-6y, whereas apigenin showed markedly higher relative abundance at AAF-2y, reaching its highest level during the entire aging period. Kaempferol, luteolin, rutin, ferulic acid, and isofraxidin showed higher relative abundances during the middle and late aging stages; however, the relative abundances of rutin and isofraxidin fluctuated across aging years. Argyinolide H and matricarin showed low relative abundances in FA and higher values in the AAF-1y to AAF-3y groups. Both compounds showed lower relative abundances in the AAF-4y to AAF-6y groups, with higher values in AAF-7y than in AAF-6y. Overall, the nine candidate metabolites did not show a consistent, unidirectional change during aging; instead, they displayed different change patterns, including early-stage higher relative abundance, marked increases at the middle stage, and sustained relative enrichment during the middle and late stages. Among these patterns, the AAF-4y represented a stage with pronounced compositional differences in this dataset, with higher relative abundances for metabolites. These findings further demonstrate that different classes of chemical constituents exhibit distinct aging-duration-associated abundance patterns among the investigated AAF samples, providing a basis for subsequent validation of candidate chemical markers.

## 3. Discussion

### 3.1. Diversity and Chemical Composition of AAF Non-Volatile Metabolites

In this study, a total of 138 metabolites were putatively annotated via UPLC-Orbitrap-MS/MS in positive-ion mode, among which flavonoids represented the most dominant annotated chemical class (47 of 138 metabolites). Consistent with previous studies, AAF is rich in diverse flavonoid components such as luteolin, apigenin, eupatilin, and jaceosidin, which jointly constitute a complex and active flavonoid metabolic network [27]. Accumulating pharmacological evidence has indicated that total flavonoids and individual flavonoids in AAF possess potent antioxidant and anti-inflammatory capacities, which exert biological functions by alleviating oxidative stress and suppressing the secretion of pro-inflammatory cytokines [28,29]. Furthermore, previous quantification of total flavonoids in AAF with different aging durations confirmed that prolonged aging increases flavonoid accumulation, further suggesting that aging duration substantially reshapes the chemical composition of AAF [30]. In addition to flavonoids, abundant terpenoids and phenolic acids were also identified in the present study, further highlighting the high compositional complexity of AAF non-volatile metabolites. Previous studies have also shown that sesquiterpenes, triterpenes, and caffeoylquinic acid derivatives are core bioactive constituents of AAF, exhibiting multiple pharmacological properties including antioxidant, anti-inflammatory, and hypoglycemic activities both in vitro and in vivo [31,32]. Therefore, these previous findings strongly support the predominance of flavonoids, phenolic acids, and terpenoids observed in this study.

While most previous studies have focused on the qualitative and quantitative identification of *Artemisia* species-derived compounds, the present study extends previous findings by systematically comparing non-volatile metabolomic profiles of AAF samples representing consecutive aging durations from 0 to 7 years, rather than merely identifying chemical compounds. Furthermore, AAF contains a variety of other metabolites, including nitrogen-containing compounds, lipids, and organic acids. Although these components are less represented in the annotated dataset, they further enrich its chemical diversity and may contribute to its overall biological efficacy.

### 3.2. Temporal Patterns of AAF Metabolomics Across Aging Duration

Multiple statistical approaches, including PCA and PLS-DA, consistently demonstrated that the overall non-volatile metabolic profiles of AAF differed among aging-duration groups. Freshly harvested AAF exhibited a distinct metabolic signature compared with aged AAF samples. At the same time, AAF samples representing 1–3 years and 4–7 years of aging formed two relatively independent clusters, suggesting stage-associated differences in their metabolic profiles. Nevertheless, the distinct separation between AAF-3y and AAF-4y samples can only represent a cross-sectional difference, rather than definitive evidence of a strict three-year aging threshold.

In pairwise difference analysis based on |log_2_ FC| > 1 and FDR < 0.01, compared with FA, 41, 55, 36, 54, 24, 36, and 60 differential metabolites were identified in AAF-1y, AAF-2y, AAF-3y, AAF-4y, AAF-5y, AAF-6y, and AAF-7y, respectively. Rather than increasing progressively with aging duration, the number of differential metabolites exhibited a non-monotonic pattern, with the largest number of differential metabolites relative to FA observed in the AAF-7y group. Pairwise comparisons among the aged samples further revealed pronounced stage-dependent variation. Notably, this study identified 57 differential metabolites between the AAF-3y and AAF-4y samples, and multivariate statistical analysis confirmed a clear metabolic separation between the two groups. These results indicate that AAF undergoes significant compositional changes between the 3-year and 4-year aging-duration groups. However, this cross-sectional difference should not be interpreted as definitive evidence of an aging-induced metabolic transition or a critical aging threshold, because the effects of harvest year and aging duration cannot be independently separated in the present sampling design. Future studies with independent batch samples, denser sampling intervals within the 3–4-year aging range, and targeted quantitative verification using reference standards are required to validate the reproducibility of this compositional shift and explore its corresponding changes in biological activity.

Further screening identified 48 differential metabolites that met the criteria of VIP ≥ 1, |log_2_ FC| > 1, and FDR < 0.01. The 48 differential metabolites formed three distinct temporal clusters, indicating that AAF samples with different aging durations are highly metabolite-specific and stage-dependent. These changes mainly included an overall decline, higher relative abundances in the middle-to-late and early aging-duration groups, rather than a uniform or monotonic trend. Similar staged compositional changes have also been documented in previous AAF aging research. For example, a multi-omics study on 0–5-year-aged AAF reported differential structural remodeling of polysaccharides during early and late aging stages, consistent with the staged metabolic trajectories observed in the present study, despite differences in research objects and analytical dimensions [33]. Another study on combustion emissions of AAF aged for different periods also found that chemical discrepancies among samples did not continuously increase with aging duration [34]. Although these previous studies focused on polysaccharides and combustion products rather than small-molecule non-volatile metabolites, their conclusions collectively support our finding that AAF chemical changes during aging duration do not follow a linear trajectory. Overall, the relative abundances of the differential metabolites varied non-monotonically among the investigated aging-duration groups, with distinct patterns across metabolite classes.

### 3.3. Relative Peak Abundance of Exploratory Candidate Markers Across Aging-Duration Groups

From the 48 differential metabolites, nine representative compounds exhibiting distinct temporal trends were selected as exploratory candidate markers associated with aging duration. Metabolites from different chemical classes displayed complementary temporal response patterns, including overall decline, mid-to-late-stage enrichment, and early-stage enrichment, suggesting that a single compound may be insufficient to fully characterize the chemical differences observed among AAF samples with different aging durations. Consistent with our findings, previous AAF aging studies have shown that samples with different aging durations can be effectively distinguished by time-dependent characteristic compounds [35]. However, the differential metabolite profiles identified in this study differ from those identified in the aforementioned studies, with only a small number of common metabolites (such as isofraxidin). Furthermore, the differential metabolites identified in this study encompass a wide range of representative chemical constituents of AAF, including flavonoids (kaempferol, apigenin, luteolin, and rutin), coumarins, phenolic acids, and terpenoids. In addition, targeted quantification has been used to investigate associations between phenolic acids, flavonoids, and antioxidant activity in AAF [36]. Compared with single-marker evaluation, a combination of the exploratory candidate markers identified here may better describe the complex, non-monotonic chemical changes during AAF aging, and merits evaluation for aging-stage discrimination [37]. Nevertheless, these exploratory candidate markers require further systematic validation before they can be used as validated markers for determining AAF aging duration.

This study characterizes aging-associated differences in AAF non-volatile metabolites and identifies exploratory candidate markers for further evaluation. However, the correlation between these exploratory candidate markers and the traditional “aged AAF moxa superiority”, as well as their underlying pharmacodynamic mechanisms, requires further experimental validation.

## 4. Materials and Methods

### 4.1. Materials and Instruments

In July 2025, AAF samples with aging periods ranging from 0 to 7 years were obtained from Nanyang Wancaotang Pharmaceutical Co., Ltd. in Henan (Nanyang, China). The leaves were harvested annually around the Dragon Boat Festival from 2018 to 2025 in the same region. After removing impurities and soil, they were air-dried naturally for 10–15 days until constant weight was achieved. They were packaged in double-layered kraft paper bags and aged in a warehouse at 18–26 °C and 50–70% relative humidity, protected from direct sunlight and turned periodically. All samples were transported to South China Botanical Garden, Chinese Academy of Sciences (Guangzhou, China). All samples were identified by Dr. Qiaoli Wang and stored in a cool, dry, dark environment to avoid light-induced degradation. LC–MS-grade acetonitrile and methanol were purchased from Fisher Scientific (Pittsburgh, PA, USA). Metabolite analysis was performed using a UPLC-Orbitrap-MS/MS platform.

### 4.2. Sample Preparation for Metabolomics

For metabolite extraction, 0.1 g of each AAF sample was mixed thoroughly with 1 mL of methanol, corresponding to a solid-to-solvent ratio of 1:10 (*w*/*v*). Accordingly, p-chlorophenylalanine was added as an internal standard with a final concentration of 1 mg/L. The mixture was ultrasonicated for 30 min and vortex-mixed repeatedly to ensure sufficient metabolite extraction. After centrifugation at 12,000 rpm for 15 min at 4 °C, 900 μL of the supernatant was transferred into an autosampler vial for subsequent UPLC-Orbitrap-MS/MS analysis. The internal standard was included to monitor analytical consistency, particularly variation in injection and LC–MS signal response. Three biologically independent samples were included for each of the eight aging-duration groups. One pooled QC sample was injected after every five biological samples. A solvent blank was included in the analytical sequence to monitor background signals and potential residual effects from samples. Quality control (QC) samples were prepared by taking equal volumes of 100 μL from each of the 24 samples, representing eight aging-duration groups (*n* = 3 per group).

### 4.3. UPLC-Orbitrap-MS/MS Analysis

All UPLC–Orbitrap–MS/MS analyses were performed on an UltiMate 3000 UPLC system coupled with an Orbitrap Elite high-resolution mass spectrometer (Thermo Fisher Scientific, Waltham, MA, USA). MS/MS spectra were acquired using higher-energy collisional dissociation (HCD) fragmentation at 60 eV. The mobile phase consisted of water containing 0.1% formic acid (Solvent A) and pure acetonitrile (Solvent B). The column flow rate was set at 0.25 mL/min, and the column temperature was maintained at 40 °C with an injection volume of 2 μL. The linear gradient elution program was optimized as follows: 0–20 min, 10–100% B; 20–25 min, 100% B; 25.1–30 min, 10% B for column re-equilibration. Chromatographic separation was performed using a Waters ACQUITY UPLC HSS T3 column (100 mm × 2.1 mm, 1.8 μm). Mass spectrometric detection was performed using an LTQ-Orbitrap Elite high-resolution mass spectrometer equipped with an electrospray ionization source operating in positive-ion mode. Instrumental parameters were set as follows: spray voltage of 3.50 kV, ion source heater temperature of 300 °C, capillary temperature of 275 °C, sheath gas flow rate of 35 arb, and auxiliary gas flow rate of 10 arb. The mass scan range was *m*/*z* 100–2000 in positive-ion mode. All UPLC-Orbitrap-MS/MS raw data were acquired using Xcalibur 4.1 software (Thermo Fisher Scientific).

### 4.4. Metabolomic Data Preprocessing and Multivariate Statistical Analysis

Raw UPLC-Orbitrap-MS/MS data were processed using Compound Discoverer 3.2 software (Thermo Fisher Scientific) for untargeted metabolomics analysis, including retention time alignment, peak detection, feature grouping, and gap filling. The retention time and mass tolerances were set to 0.2 min and 5 ppm, respectively. Metabolite annotation confidence was reported using the Metabolomics Standards Initiative (MSI) framework. Metabolites were putatively annotated by comparing accurate precursor masses and experimental MS/MS spectra against data from publicly available databases (HMDB, PubChem, ChemSpider, and mzCloud) and against fragmentation information for metabolites reported in the literature. Prior to multivariate analysis, the peak area matrix was transformed using log_2_(x) and standardized using Pareto scaling for subsequent statistical analysis.

Multivariate statistical analysis was performed in R. Principal component analysis (PCA) was employed to evaluate the overall metabolic profiles, sample distribution, and intragroup reproducibility of AAF samples aged for different durations [38]. PCA score plots based on the first two principal components (PC1 and PC2) were used to visualize sample clustering, trends in group separation, and the distribution characteristics of quality control (QC) samples [39]. Subsequently, partial least squares discriminant analysis (PLS-DA) was used to assess metabolic differences among samples at different aging stages, and the model’s stability and statistical reliability were evaluated using 100 repeats of stratified three-fold cross-validation and 1000-permutation tests. PLS-DA models containing 1–5 components were compared using 100 repetitions of stratified three-fold cross-validation, and the model with 4 components was retained. The significance of the model was assessed using a 1000-permutation test. In addition, based on the Pareto scaling matrix and Euclidean distances of 24 samples, PERMANOVA and PERMDISP tests were performed using the vegan package to evaluate overall between-group differences and multivariate dispersion, respectively, with 9999 permutations in each case. Using aging duration (0–7 years) as the continuous response variable, a PLS regression model was constructed using the ropls package, which automatically determined the number of components. Model performance and significance were evaluated using three-fold cross-validation and 1000 permutations. Finally, variable importance in projection (VIP) values were calculated to measure the contribution of metabolite features to intergroup classification, and metabolic features with VIP ≥ 1 were identified as key differential variables [40,41,42].

All 28 pairwise comparisons were performed across the eight aging-duration groups. For each metabolite, the log_2_-fold change (log_2_ FC) was calculated from relative abundances before log_2_ transformation. Intergroup differences were evaluated using a two-sided Welch’s *t*-test based on log_2_-transformed relative abundances. For each pairwise comparison, the raw *p*-values for all putatively annotated metabolites were treated as an independent family of multiple tests and uniformly corrected using the Benjamini–Hochberg method to control the false discovery rate (FDR); thus, each pairwise comparison underwent independent FDR correction across the putatively annotated metabolites. Metabolites with |log_2_ FC| > 1 and FDR-adjusted *p* < 0.01 were selected as differential metabolites and visualized using volcano plots and heatmaps [43]. Mfuzz clustering was used to characterize the abundance patterns of 48 preselected differential metabolites across different aging-duration groups. Prior to clustering, the log_2_-transformed relative abundances from three biological replicates in each group were averaged and normalized. The number of candidate clusters was set to 2–8, and three clusters were ultimately determined based on the minimum centroid distance (Dmin). The Mfuzz (v 2.70.0) results were visualized using temporal trend plots; metabolites were ordered within each Mfuzz cluster using hierarchical clustering with Euclidean distance and complete linkage. For the heatmap, all 24 samples were retained without within-group averaging, and row-wise Z-score normalization was applied to each metabolite before plotting [44]. All data processing, statistical analyses, and graphical visualization were performed using R software (v 4.5.2; R Core Team, Vienna, Austria).

### 4.5. Exploratory Candidate Markers Selection and Abundance Visualization

Exploratory candidate markers were selected from the differential metabolites based on their previously reported occurrence in AAF, their representation of different chemical classes, and their coverage of relative abundance patterns. No additional selection thresholds, numerical rankings, or composite scores were applied. These metabolites were therefore selected as representative exploratory candidate markers associated with aging duration [45]. Finally, the patterns of relative abundance changes for the relevant exploratory candidate markers across different aging duration groups were visualized to illustrate their changes over years of aging in AAF.

## 5. Conclusions

This study used UPLC-Orbitrap-MS/MS-based untargeted metabolomics in positive ion mode to characterize non-volatile metabolites in eight AAF sample groups representing different aging durations from 0 to 7 years. A total of 138 metabolites were putatively annotated, and 48 differential metabolites showed three major aging-associated patterns. Nine compounds, comprising four flavonoids, two coumarins, two terpenoids, and one phenolic acid, were selected as exploratory candidate markers. The overall non-volatile metabolic profiles of AAF samples with different aging durations exhibited marked differences. These results indicate that the relative abundances of non-volatile metabolites in AAF samples representing different aging durations exhibit complex, non-monotonic patterns rather than a consistent, unidirectional trend. Collectively, this study characterizes the compositional differences among AAF samples with different aging histories and provides an exploratory chemical dataset and a preliminary set of candidate metabolites for future investigations into AAF batch differentiation and aging-duration-associated compositional variation. Future investigations should incorporate identity confirmation using authentic standards, absolute quantification, and pharmacological activity evaluation to further clarify the quantitative relationship between exploratory candidate markers and aging duration, aiming to upgrade the current AAF aging evaluation strategy from holistic metabolic profiles to reliable, verifiable chemical quality assessment.

## Figures and Tables

**Table 1:** Putative annotations and MS/MS features of nine exploratory candidate markers associated with AAF at different aging durations, as determined in positive-ion mode.

No.	*t*_R_ (min)	Molecular Formula	Adduct Ion	Measured *m*/*z*	Theoretical *m*/*z*	Mass Error (ppm)	Component Name	MS/MS Fragments (HCD)	Compound Classification	Confidence Level
1	4.92	C_9_H_6_O_4_	[M + H]^+^	179.0343	179.0339	2.23	Esculetin	179.0344, 123.0444, 133.0288, 135.0445, 151.0394	Lignans and coumarins	2
2	5.64	C_27_H_30_O_16_	[M + H]^+^	611.1625	611.1607	2.95	Rutin	85.0285, 303.0507	Flavonoids	2
3	6.37	C_11_H_10_O_5_	[M + H]^+^	223.0604	223.0601	1.35	Isofraxidin	162.0317, 190.0267, 207.0295, 223.0608	Lignans and coumarins	2
4	6.48	C_15_H_10_O_6_	[M + H]^+^	287.0557	287.0550	2.44	Luteolin	137.0240, 151.0190, 213.0557, 258.0535, 287.0563	Flavonoids	2
5	8.02	C_20_H_28_O_6_	[M + H]^+^	365.1963	365.1959	1.10	Argyinolide H	57.0699, 109.0284, 227.1067, 245.1172, 263.1278, 281.1384, 323.1489, 347.1853, 365.1959	Terpenoids	2
6	9.02	C_10_H_10_O_4_	[M + H − H_2_O]^+^	177.0551	177.0546	2.82	Ferulic acid	117.0332, 134.0371, 145.0283, 177.0545	Phenolic acids	2
7	9.56	C_15_H_10_O_6_	[M + H]^+^	287.0554	287.0550	1.39	Kaempferol	165.0190, 213.0558, 287.0559, 258.0525	Flavonoids	2
8	10.61	C_17_H_20_O_5_	[M + H]^+^	305.1399	305.1384	4.92	Matricarin	69.0327, 245.1165, 217.1208, 305.1384	Terpenoids	2
9	12.21	C_15_H_10_O_5_	[M + H]^+^	271.0606	271.0601	1.84	Apigenin	153.0187, 271.0607	Flavonoids	2

Notes: Data are numbered according to retention time (tR). MSI level: confidence levels as defined by the Metabolomics Standards Initiative [18]: Level 2—putatively annotated based on spectral similarity and diagnostic fragments.

## Data Availability

Data of supporting the results are included in this manuscript and its Supplementary Materials. Further inquiries can be directed to the corresponding author.

## Supplementary Materials

The following supporting information can be downloaded at https://www.mdpi.com/article/10.3390/molecules31213995/s1. Figure S1. Total ion chromatograms (TICs) of AAF samples with different aging durations in positive ion mode: (a) FA (freshly harvested AAF, 0 years); (b) AAF-1y; (c) AAF-2y; (d) AAF-3y; (e) AAF-4y; (f) AAF-5y; (g) AAF-6y; (h) AAF-7y. Retention time (min) is shown on the x-axis and relative abundance (%) on the y-axis. Figure S2. Evaluation of the classification accuracy of PLS-DA models with 1–5 components using 100 repetitions of stratified three-fold cross-validation. Points represent the mean accuracy across the resulting 300 validation folds. Error bars indicate ±1 standard deviation (SD) across fold-level accuracies, with bounds truncated to [0, 1]. The vertical dashed line indicates the four-component model adopted for the final analysis. Figure S3. Diagnostic evaluation of the final four-component PLS-DA model for eight groups of AAF samples with different aging durations. (a) Cumulative model-fitting and cross-validated predictive parameters obtained with increasing numbers of predictive components. The gray and black bars represent cumulative R2Y and Q2, respectively; (b) Results of the 1000-permutation test. The original model showed significantly higher R2Y and Q2 values than the randomly permuted models (*p* = 0.001); (c) Observation diagnostic plot showing the score distance and orthogonal distance of each sample. The dashed lines indicate the model-derived diagnostic limits used to identify potentially unusual observations; (d) Score plot based on the first two predictive components of the final four-component model. The model yielded cumulative R2X = 0.80, R2Y = 0.57, and Q2 = 0.56, with a root mean squared error of estimation (RMSEE) of 0.24. Figure S4. Volcano plots of differential metabolites between FA and AAF samples stored for 1–7 years. The individual subplots represent: (a) FA vs. AAF-1y, (b) FA vs. AAF-2y, (c) FA vs. AAF-3y, (d) FA vs. AAF-4y, (e) FA vs. AAF-5y, (f) FA vs. AAF-6y, (g) FA vs. AAF-7y. Figure S5. Volcano plots of differential metabolites between AAF-1y and AAF samples stored for 2–7 years. The subplots represent, respectively: (a) AAF-1y vs. AAF-2y, (b) AAF-1y vs. AAF-3y, (c) AAF-1y vs. AAF-4y, (d) AAF-1y vs. AAF-5y, (e) AAF-1y vs. AAF-6y, and (f) AAF-1y vs. AAF-7y. Figure S6. Volcano plots of differential metabolites between AAF-2y and AAF samples stored for 3–7 years. The subplots represent, respectively: (a) AAF-2y vs. AAF-3y, (b) AAF-2y vs. AAF-4y, (c) AAF-2y vs. AAF-5y, (d) AAF-2y vs. AAF-6y, and (e) AAF-2y vs. AAF-7y. Figure S7. Volcano plots of differential metabolites between AAF-3y and AAF samples stored for 4–7 years. The subplots represent, respectively: (a) AAF-3y vs. AAF-4y, (b) AAF-3y vs. AAF-5y, (c) AAF-3y vs. AAF-6y, and (d) AAF-3y vs. AAF-7y. Figure S8. Volcano plots of differential metabolites between AAF-4y and AAF samples stored for 5–7 years. The subplots represent, respectively: (a) AAF-4y vs. AAF-5y, (b) AAF-4y vs. AAF-6y, and (c) AAF-4y vs. AAF-7y. Figure S9. Volcano plots of differential metabolites between AAF-5y and AAF samples stored for 6 and 7 years. The subplots represent, respectively: (a) AAF-5y vs. AAF-6y, (b) AAF-5y vs. AAF-7y. Figure S10. Volcano plot of differential metabolites between AAF-6y and AAF-7y. Figure S11. Internal cross-validation of the four-component PLS-DA model. (a) Aggregated out-of-fold prediction confusion matrix, with rows and columns representing true and predicted classes, respectively; percentages in parentheses indicate row percentages; (b) recall rates for each class; (c) mean ± SD of accuracy, balanced accuracy, and Macro-F1. Results are from 100 repetitions of stratified three-fold cross-validation; error bars represent the standard deviation across 300 test folds, truncated to [0, 1]. The 2400 prediction records were derived from duplicate tests of 24 samples. Figure S12. (a) Aggregated confusion matrix of outer-layer predictions, where rows and columns represent true and predicted classes, respectively; percentages in parentheses indicate row percentages; (b) Recall rates for each class; (c) Component selection frequencies. The outer layer employed 100 repetitions of stratified three-fold cross-validation, and within each outer-layer training set, 10 repetitions of stratified two-fold cross-validation were used to select the number of components. For models with 1–5 components, the model with the fewest components and an average inner-layer accuracy differing by no more than 1 percentage point from the maximum value was selected. The 2400 prediction records were derived from replicate tests of 24 biological samples (3 per group), with each sample tested 100 times. These results evaluate the component selection process rather than a fixed four-component model. FA denotes fresh AAF, while AAF-1y–AAF-7y denotes samples aged 1–7 years. Figure S13. Principal coordinate analysis (PCoA) of the non-volatile metabolic profiles of AAF samples across aging-duration groups. Euclidean distances were calculated from the Pareto-scaled data matrix. Circles represent individual biological samples (*n* = 3 per group); crosses indicate group centroids, and lines connect samples to their respective centroids. PCoA1 and PCoA2 explain 31.8% and 27.5% of the variation, respectively. Overall group differences were assessed using PERMANOVA with 9999 permutations (F = 30.613, R2 = 0.9305, *p* = 0.0001). Figure S14. Homogeneity of multivariate dispersions among AAF aging-duration groups. Colored circles represent individual sample distances to the spatial median of their respective groups. Open diamonds and error bars indicate group means ± SE (*n* = 3 per group). Multivariate dispersion was assessed using PERMDISP with 9999 permutations (F = 1.865, *p* = 0.1390). Figure S15. Diagnostic evaluation of the PLS regression model for eight groups of AAF samples with different aging durations. (a) Cumulative R2Y and cross-validated Q2 for models with one to five predictive components; gray and black bars represent R2Y and Q2, respectively; (b) Results of the 1000-permutation test (*p* = 0.001 for both R2Y and Q2); (c) Observation diagnostic plot showing the score distance and orthogonal distance of each sample. The dashed lines indicate the model-derived diagnostic limits used to identify potentially unusual observations; (d) Score plot based on the first two predictive components of the five-component PLS regression model. The model yielded cumulative R2X = 0.77, R2Y = 0.96, and Q2 = 0.92, with a root mean squared error of estimation (RMSEE) of 0.95. Figure S16. Workflow of UPLC-Orbitrap-MS/MS-based metabolomic analysis and exploratory candidate markers selection in AAF samples with different aging durations. Figure S17. Distribution of feature relative standard deviations (RSDs) values across all QC samples. Table S1: Putatively annotated non-volatile metabolites in AAF samples representing different aging durations in positive-ion mode. Table S2: Compound Discoverer workflow parameter settings. Table S3: UPLC–Orbitrap MS/MS acquisition parameters recorded in raw data files.

## References

[1] Chinese Pharmacopoeia Commission (2025). Pharmacopoeia of the People’s Republic of China.

[2] Yang W., Huang Q., Wu M., Mei Q., Zou Y., Qian Z., Tang D. (2024). Rapid screening and evaluation of natural antioxidants from leaf, stem, and root of *Artemisia argyi* by online liquid microextraction combined with HPLC-based antioxidant assay system coupled with calibration quantitative analysis. J. Sep. Sci..

[3] Cui L., Wang X., Lu J., Tian J., Wang L., Qu J., Liu Z., Wei J. (2021). Rapid identification of chemical constituents in *Artemisia argyi* Lévi. et Vant by UPLC-Q-Exactive-MS/MS. J. Food Qual..

[4] Yu D., Huang N., Du X. (2023). Review of the chemical composition and biological activities of essential oils from *Artemisia argyi*, *Artemisia princeps*, and *Artemisia montana*. Curr. Top. Med. Chem..

[5] Chen C., Li J., Chen H., Cai H., Zhang J., Guo L., Miao Y., Liu D. (2024). Comprehensive review of botanical characteristics, artificial cultivation methods, quality evaluation, genome research, and potential applications of *Artemisia argyi* Lévl. et Van. Med. Plant Biol..

[6] Yang D., Zhou T., Niu T., Yang X., Fu G., Song C., Hou X. (2024). Regularity of *Artemisia argyi* Harvest time and its response to meteorological factors. Agronomy.

[7] Zhang L., Wei Y., Wang W., Fan Y., Li F., Li Z., Lin A., Gu H., Song M., Wang T. (2023). Quantitative fingerprint and antioxidative properties of *Artemisia argyi* leaves combined with chemometrics. J. Sep. Sci..

[8] Guo D., Yang Y., Wu Y., Liu Y., Cao L., Shi Y., Wan N., Wu Z. (2023). Chemical composition analysis and discrimination of essential oils of *Artemisia argyi* folium from different germplasm resources based on electronic nose and GC/MS combined with chemometrics. Chem. Biodivers..

[9] Zhang L., Wang X., Wang W., Ning E., Chen L., Li Z., Yu L., Li X., Zong W. (2024). Metabolomic analysis reveals the changing trend and differential markers of volatile and nonvolatile components of *Artemisiae argyi* with different aging years. Phytochem. Anal..

[10] Li Y., Li Y., Fang T., Feng X., Gao D. (2025). Impact of processing methods and storage on the chemical composition of *Artemisia argyi* essential oil. Ind. Crops Prod..

[11] Liu Y., He Y., Hu Q., Yang X., Ma H., Huang H., Yang M., Zhang D. (2025). Storage process: A new method reduces the acute toxicity of the essential oil of *Artemisia argyi* H. Lév. & vaniot by 40%. Future Integr. Med..

[12] Guo X., Wang D., Xiao Y., Cao H., Yao D., Chen G., Li S., Wang G., Tu J., Liu Y. (2024). Artificial aging conditions for *Artemisia argyi* leaves based on quality-inflammation-quality marker transformation. Phytomedicine.

[13] Li M., Chai X., Wang L., Yang J., Wang Y. (2019). Study of the variation of phenolic acid and flavonoid content from fresh *Artemisiae argyi* folium to moxa wool. Molecules.

[14] Gong M., Lu J.Q. (2019). Studies on total flavonoids and total polysaccharides of reserved folium *Artemisia argyi* in different years. Hubei Agric. Sci..

[15] Bao X., Yuan H., Wang C., Liu J., Lan M. (2013). Antitumor and immunomodulatory activities of a polysaccharide from *Artemisia argyi*. Carbohydr. Polym..

[16] Han B., Xin Z., Ma S., Liu W., Zhang B., Ran L., Yi L., Ren D. (2017). Comprehensive characterization and identification of antioxidants in folium *Artemisiae argyi* using high-resolution tandem mass spectrometry. J. Chromatogr. B.

[17] Yang J., Xu Y., Feng Y., Liu Q., Li M., Yang Z., Huang Z., Wan S., Wang Q., Zhang X. (2026). Effect of aging process on the structural features and immunomodulatory activity of *Artemisia argyi* polysaccharides. Chem. Biol. Technol. Agric..

[18] Alseekh S., Aharoni A., Brotman Y., Contrepois K., D’Auria J., Ewald J., Ewald J.C., Fraser P.D., Giavalisco P., Hall R.D. (2021). Mass spectrometry-based metabolomics: A guide for annotation, quantification and best reporting practices. Nat. Methods.

[19] Cui Y., Pan D., Feng J., Zhao D., Liu M., Dong Z., Liu S., Wang S. (2024). Untargeted metabolomics and soil community metagenomics analyses combined with machine learning evaluation uncover geographic differences in ginseng from different locations. J. Agric. Food Chem..

[20] Kang L., Zhang Y., Zhou L., Yang J., He Y., Yang S., Li G., Hao Q., Yu Y., Guo L. (2022). Structural characterization and discrimination of *Morinda officinalis* and processing *Morinda officinalis* based on metabolite profiling analysis. Front. Chem..

[21] Lu Y., Yao G., Wang X., Zhang Y., Zhao J., Yu Y.-J., Wang H. (2022). Chemometric discrimination of the geographical origin of licorice in China by untargeted metabolomics. Food Chem..

[22] Yu D., Zhang K., Wu J., Li X., Zhou G., Wan Y. (2023). Integrated metabolomic and transcriptomic analysis revealed the flavonoid biosynthesis and regulation in *Areca catechu*. Phytochem. Anal..

[23] Wu C.-Y., Guo Y.-Y., Zhou J., Long F., Zhang W., Shen H., Xu J.-D., Zhou S.-S., Li S.-L. (2022). Holistic quality evaluation of *Hibisci mutabilis* folium by integrating UPLC–QTOF–MS/MS chemical profiling and UPLC–TQ–MS/MS quantification approaches. J. Pharm. Biomed. Anal..

[24] Wu C.-Y., Kong M., Zhang W., Long F., Zhou J., Zhou S.-S., Xu J.-D., Xu J., Li S.-L. (2018). Impact of sulphur fumigation on the chemistry of ginger. Food Chem..

[25] Zhu Y.-Q., Liu J.-W., Zhou J., Wu C.-Y., Long F., Xu J.-D., Zhou S.-S., Mao Q., Kong M., Li S.-L. (2025). Impact of ultralow oxygen preservation on the quality of Atractylodis Macrocephalae Rhizoma evaluated by multi-chromatographic determination of small molecules and polysaccharides. J. Pharm. Biomed. Anal..

[26] Hu C., Xu G. (2014). Metabolomics and Traditional Chinese Medicine. TrAC Trends Anal. Chem..

[27] Lee S., Won H.J., Ban S., Park Y.J., Kim S.M., Kim H.S., Choi J., Kim H.-Y., Lee J.H., Jung J.H. (2023). Integrative analysis of metabolite and transcriptome reveals biosynthetic pathway and candidate genes for eupatilin and jaceosidin biosynthesis in *Artemisia argyi*. Front. Plant Sci..

[28] Hu Q., Liu Z., Guo Y., Lu S., Du H., Cao Y. (2021). Antioxidant capacity of flavonoids from folium *Artemisiae argyi* and the molecular mechanism in Caenorhabditis elegans. J. Ethnopharmacol..

[29] Li S., Zhou S., Yang W., Meng D. (2018). Gastro-protective effect of edible plant *Artemisia argyi* in ethanol-induced rats via normalizing inflammatory responses and oxidative stress. J. Ethnopharmacol..

[30] Lim M.Y., Huang J., Zhao B.-X., Zou H.-Q., Yan Y.-H. (2016). Influence of storage duration and processing on chromatic attributes and flavonoid content of moxa floss. J. Integr. Med..

[31] Xiao J.-Q., Liu W.-Y., Sun H.-P., Li W., Koike K., Kikuchi T., Yamada T., Li D., Feng F., Zhang J. (2019). Bioactivity-based analysis and chemical characterization of hypoglycemic and antioxidant components from *Artemisia argyi*. Bioorg. Chem..

[32] Wei W.-J., Guo T., Xue G.-M., Ma R., Wang Y., Chen Z.-S. (2024). *Artemisia argyi* H. Lév. & Vaniot: A comprehensive review on traditional uses, phytochemistry, and pharmacological activities. Phytochem. Rev..

[33] Wang X., Pan L., Wang L., Liu S., Du Y., Zhang R., Liu S., Zhang D., Wang D., Wu F. (2026). Endophytes-mediated structural remodeling of polysaccharides and small molecules in aging *Artemisia argyi* leaves. Int. J. Biol. Macromol..

[34] Zhai M., Liu J.-N., Wang L.-H., Wen Y.-J., Ma H., Liu P.-P., Chai G.-B., Zhang Q.-D., Ma J., Yu Y.-J. (2025). Evaluation of annual storage on online released compounds in *Artemisia argyi* smoke with GC-MS-based uindicate ntargeted metabolomics coupled with chemometric method of antDAS-GCMS. J. Chemom..

[35] Zhang K., Wang S., Zhang H., Ming J., Long W., Fu H. (2026). Revealing the chemical differences and their application on geographical origin and aging year identification of *Artemisia argyi* folium based on GC–MS and UHPLC-HRMS untargeted metabolomics. Food Res. Int..

[36] Xia J.-X., Zhao B.-B., Zan J.-F., Wang P., Chen L.-L. (2019). Simultaneous determination of phenolic acids and flavonoids in *Artemisiae argyi* folium by HPLC-MS/MS and discovery of antioxidant ingredients based on relevance analysis. J. Pharm. Biomed. Anal..

[37] Fan X., Long W., Lan W., Liu D., Ming J., Chen H., Fu H. (2025). Deep learning identification of *Artemisiae argyi* folium aging years and prediction of active ingredients via Fe^2+^-mediated Fenton reaction colorimetric sensor. Microchem. J..

[38] Zhang T., Wan X., Liu C., Luo J., Wan D., Yang X. (2024). A comparative UPLC-MS-based metabolomics approach for distinguishing four different cultivars of Qiai (*Artemisia argyi* Lévl. et Van.). Arab. J. Chem..

[39] Mhlongo M.I., Steenkamp P.A., Piater L.A., Madala N.E., Dubery I.A. (2016). Profiling of altered metabolomic states in nicotiana tabacum cells induced by priming agents. Front. Plant Sci..

[40] Szymańska E., Saccenti E., Smilde A.K., Westerhuis J.A. (2012). Double-Check: Validation of diagnostic statistics for PLS-DA models in metabolomics studies. Metabolomics.

[41] Thévenot E.A., Roux A., Xu Y., Ezan E., Junot C. (2015). Analysis of the human adult urinary metabolome variations with age, body mass index, and gender by implementing a comprehensive workflow for univariate and OPLS statistical analyses. J. Proteome Res..

[42] Westerhuis J.A., Hoefsloot H.C.J., Smit S., Vis D.J., Smilde A.K., Van Velzen E.J.J., Van Duijnhoven J.P.M., Van Dorsten F.A. (2008). Assessment of PLSDA cross validation. Metabolomics.

[43] Idkowiak J., Dehairs J., Schwarzerová J., Olešová D., Truong J.X.M., Kvasnička A., Eftychiou M., Cools R., Spotbeen X., Jirásko R. (2025). Best practices and tools in R and Python for statistical processing and visualization of lipidomics and metabolomics data. Nat. Commun..

[44] Li H., Ma C., Li S., Wang H., Fang L., Feng J., Wang Y., Li Z., Cai Q., Geng X. (2023). Eight typical aroma compounds of ‘panguxiang’ pear during development and storage identified via metabolomic profiling. Life.

[45] Lei H., Zhang Y., Zu X., Ye J., Liang Y., Cheng T., Zhang W. (2021). Comprehensive profiling of the chemical components and potential markers in raw and processed *Cistanche tubulosa* by combining ultra-high-performance liquid chromatography coupled with tandem mass spectrometry and MS/MS-based molecular networking. Anal. Bioanal. Chem..

