# Supplementary material for: Metabolomics-Based Analysis Reveals Differences in Non-Volatile Components of Artemisiae argyi Folium with Different Aging Durations
Source: Molecules. 2026 Nov 5;31(21):3995. doi: 10.3390/molecules31213995 (PMC13765397; doi:10.3390/molecules31213995)
Supplement: Supplementary file 1 [file molecules-31-03995-s001.zip › molecules-4617978-supplementary.pdf]

Metabolomics-Based Analysis Reveals Differences in Non-Volatile Components of *Artemisiae argyi* Folium with Different Aging Durations

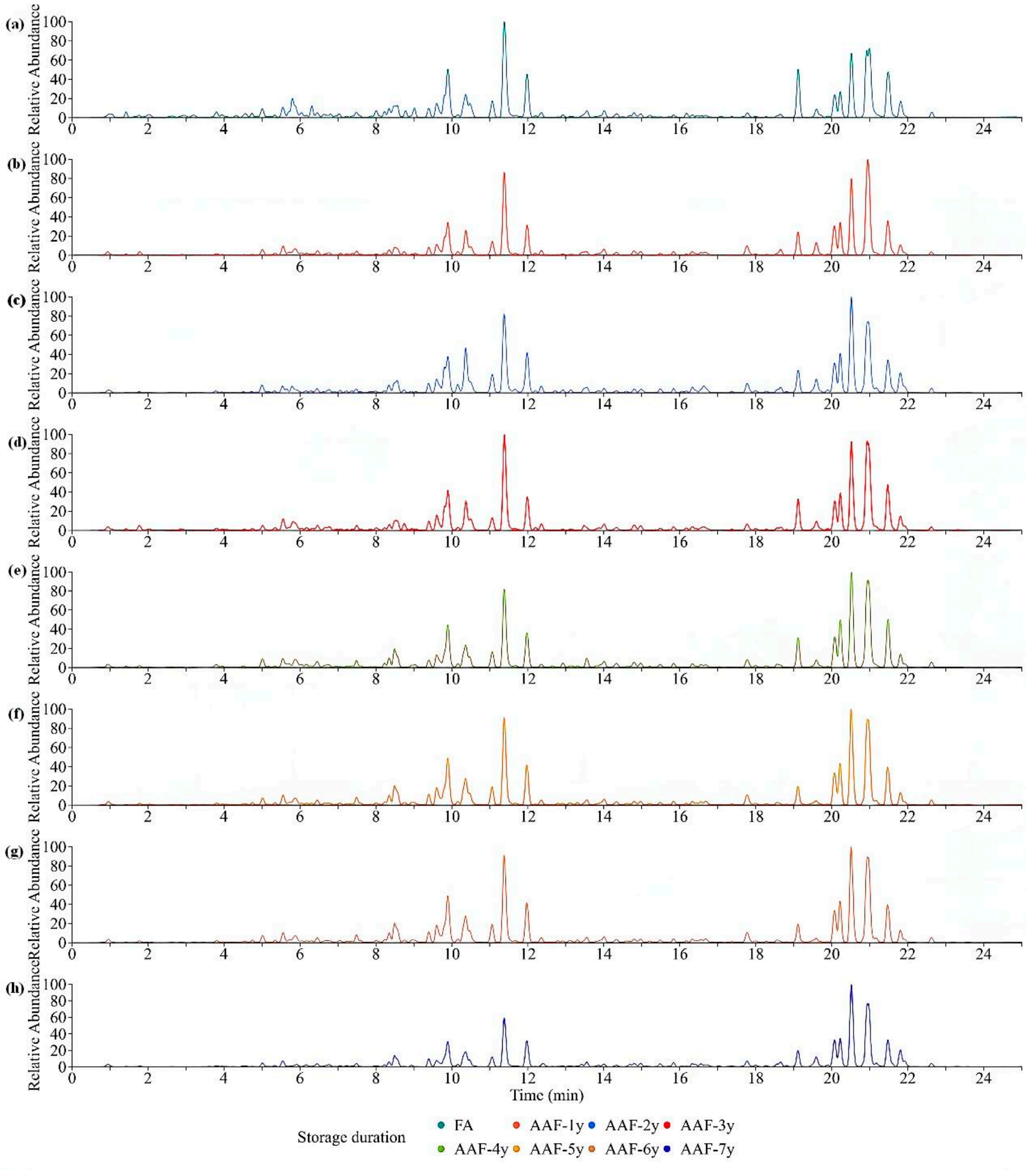

Figure S1. Total ion chromatograms (TICs) of AAF samples with different aging durations in positive ion mode: (a) FA (freshly harvested AAF, 0 years); (b) AAF-1y; (c) AAF-2y; (d) AAF-3y; (e) AAF-4y; (f) AAF-5y; (g) AAF-6y; (h) AAF-7y. Retention time (min) is shown on the x-axis and relative abundance (%) on the y-axis.

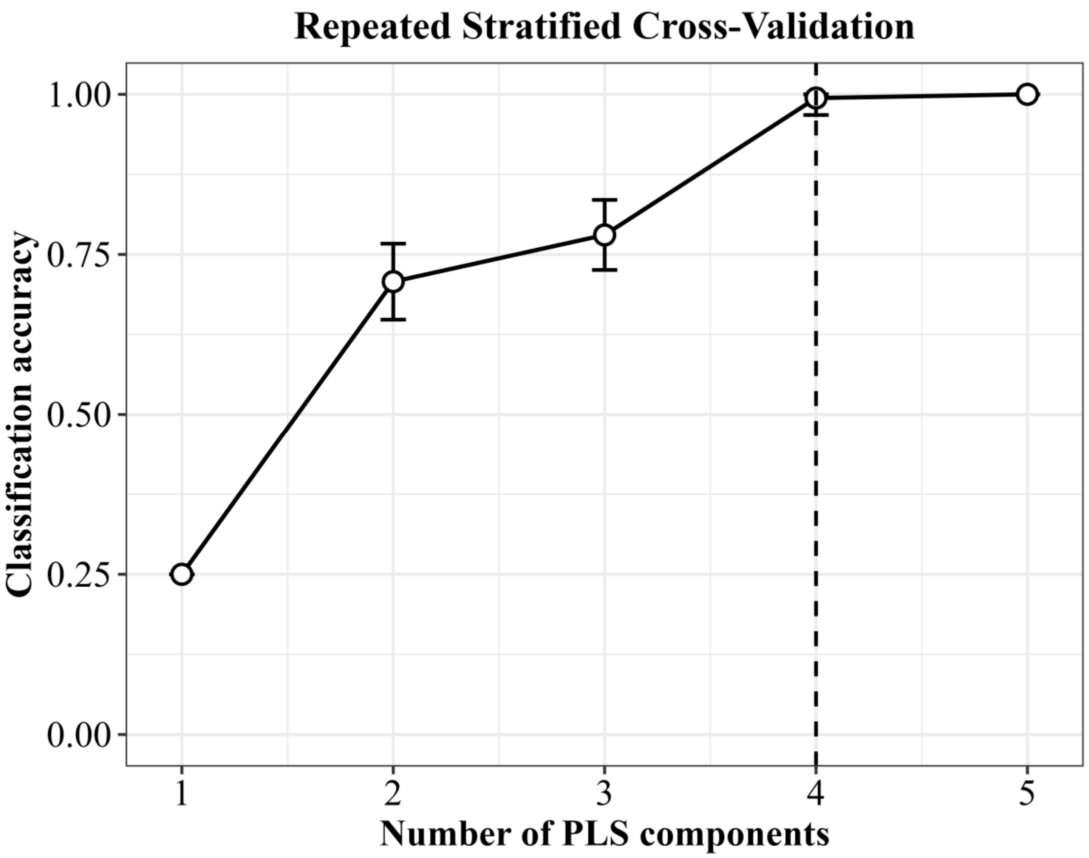

Figure S2. Evaluation of the classification accuracy of PLS-DA models with 1–5 components using 100 repetitions of stratified three-fold cross-validation. Points represent the mean accuracy across the resulting 300 validation folds. Error bars indicate ±1 standard deviation (SD) across fold-level accuracies, with bounds truncated to [0, 1]. The vertical dashed line indicates the four-component model adopted for the final analysis.

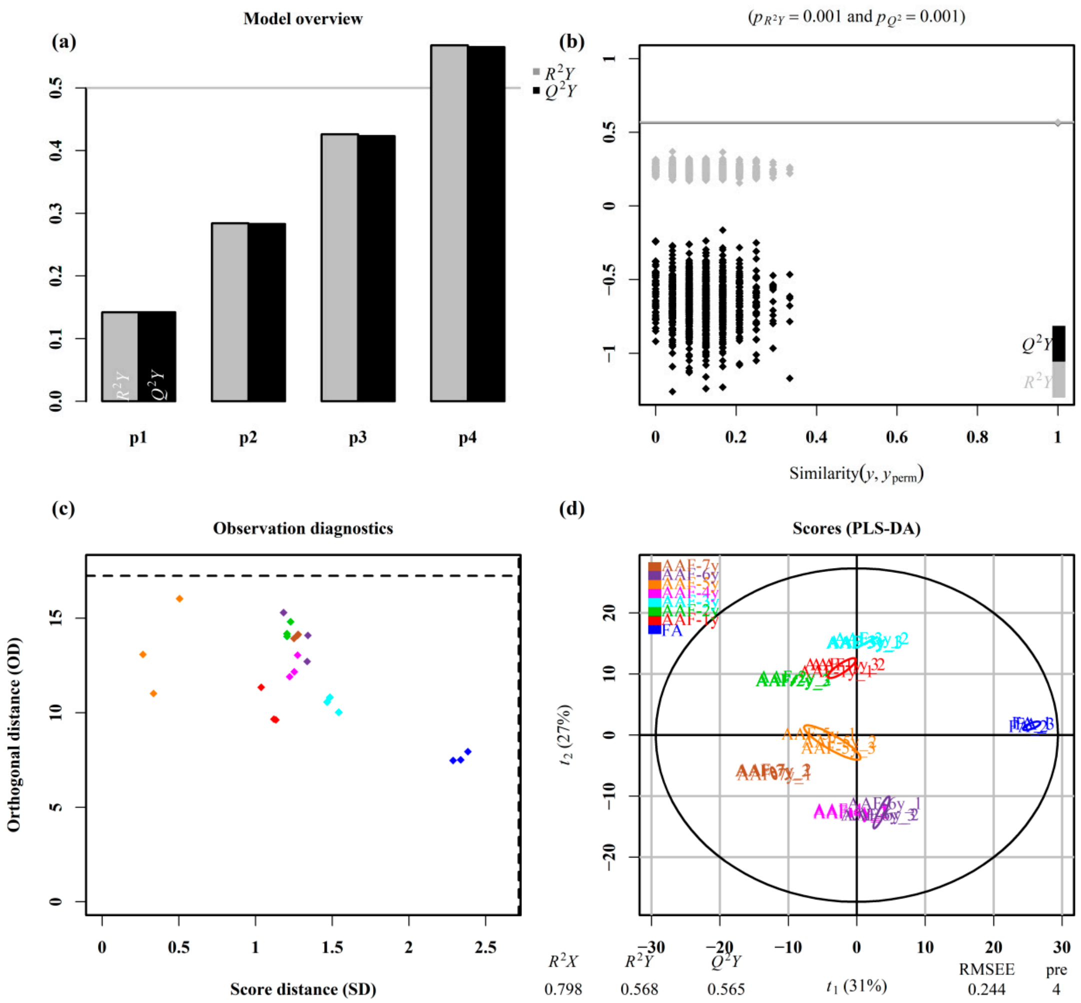

Figure S3. Diagnostic evaluation of the final four-component PLS-DA model for eight groups of AAF samples with different aging durations. (a) Cumulative model-fitting and cross-validated predictive parameters obtained with increasing numbers of predictive components. The gray and black bars represent cumulative R^2Y and $Q^{2,}$ respectively; (b) Results of the 1000-permutation test. The original model showed significantly higher R^2Y and Q^2 values than the randomly permuted models (p = 0.001); (c) Observation diagnostic plot showing the score distance and orthogonal distance of each sample. The dashed lines indicate the model-derived diagnostic limits used to identify potentially unusual observations; (d) Score plot based on the first two predictive components of the final four-component model. The model yielded cumulative R^2X = 0.80, R^2Y = 0.57, and Q^2 = 0.56, with a root mean squared error of estimation (RMSEE) of 0.24.

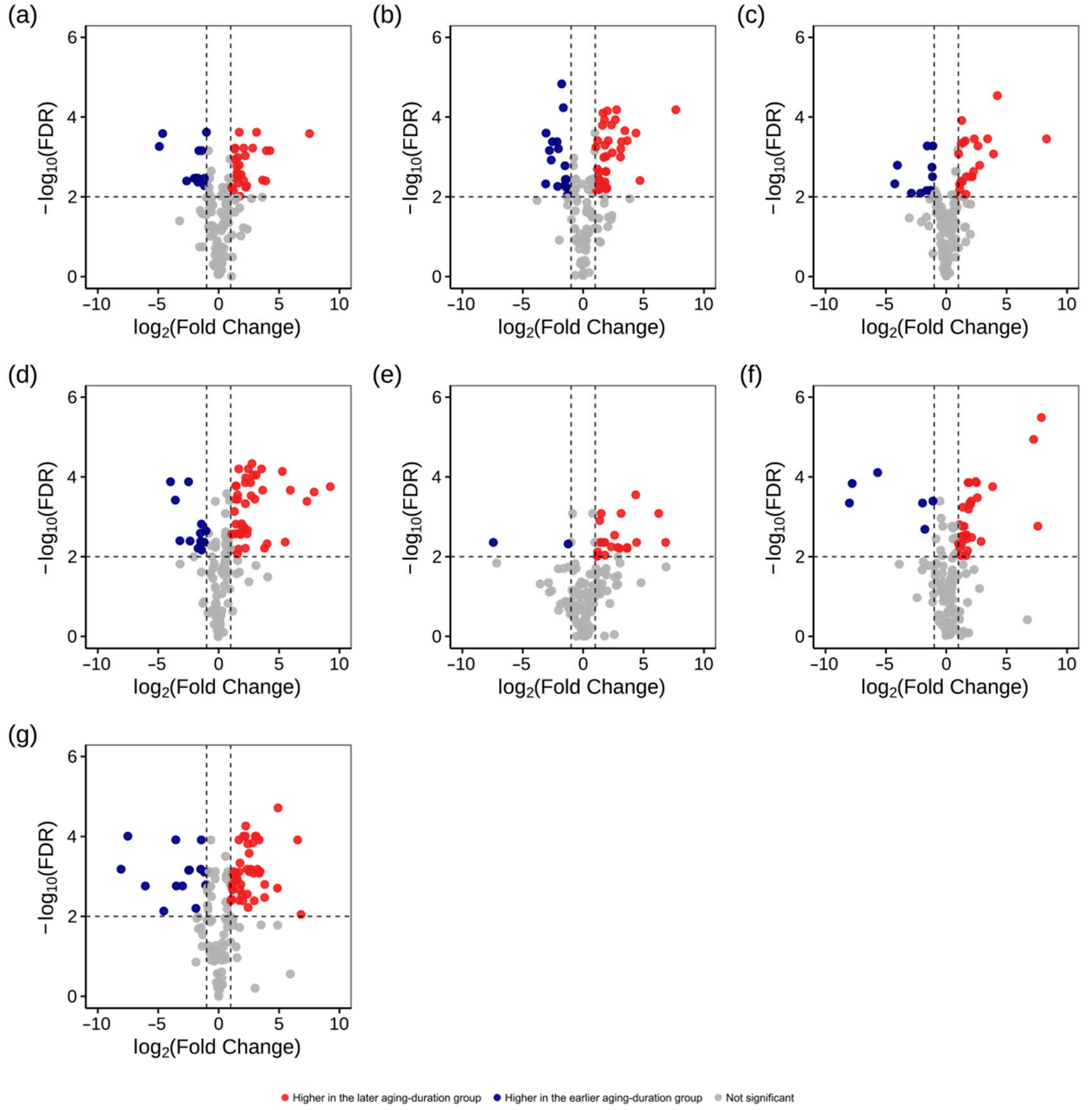

Figure S4. Volcano plots of differential metabolites between FA and AAF samples stored for 1–7 years. The individual subplots represent: (a) FA vs. AAF-1y, (b) FA vs. AAF-2y, (c) FA vs. AAF-3y, (d) FA vs. AAF-4y, (e) FA vs. AAF-5y, (f) FA vs. AAF-6y, (g) FA vs. AAF-7y.

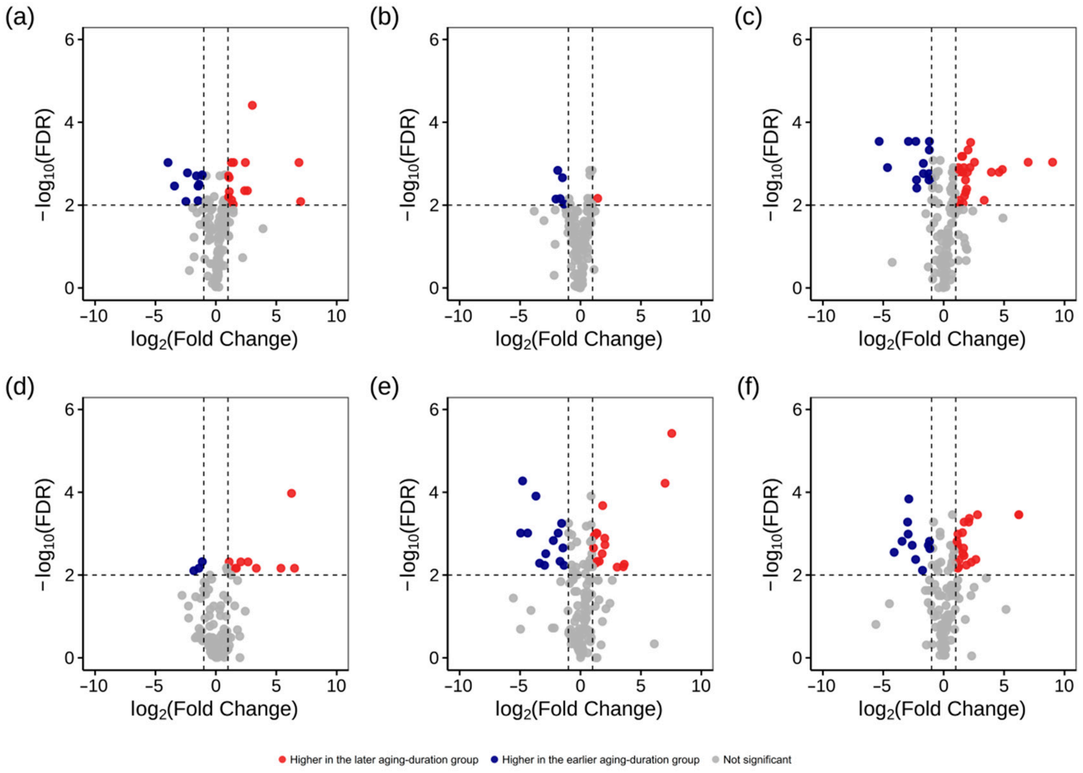

Figure S5. Volcano plots of differential metabolites between AAF-1y and AAF samples stored for 2–7 years. The subplots represent, respectively: (a) AAF-1y vs. AAF-2y, (b) AAF-1y vs. AAF-3y, (c) AAF-1y vs. AAF-4y, (d) AAF-1y vs. AAF-5y, (e) AAF-1y vs. AAF-6y, and (f) AAF-1y vs. AAF-7y.

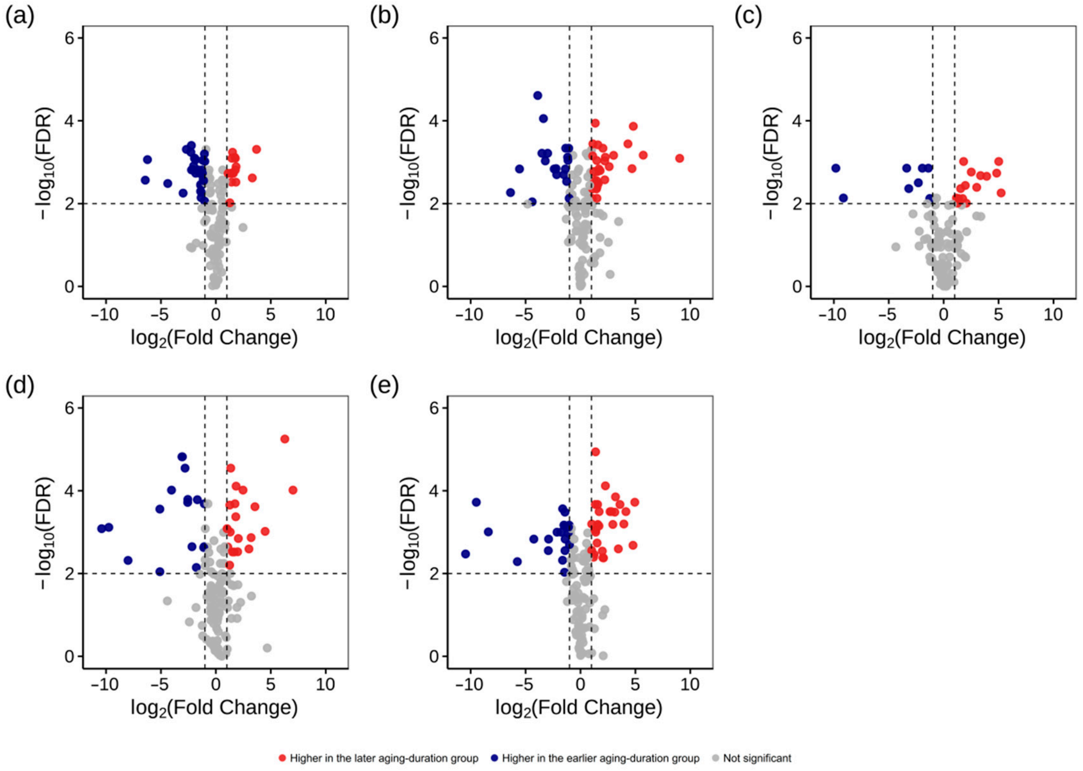

Figure S6. Volcano plots of differential metabolites between AAF-2y and AAF samples stored for 3–7 years. The

subplots represent, respectively: (a) AAF-2y vs. AAF-3y, (b) AAF-2y vs. AAF-4y, (c) AAF-2y vs. AAF-5y, (d) AAF-2y vs. AAF-6y, and (e) AAF-2y vs. AAF-7y.

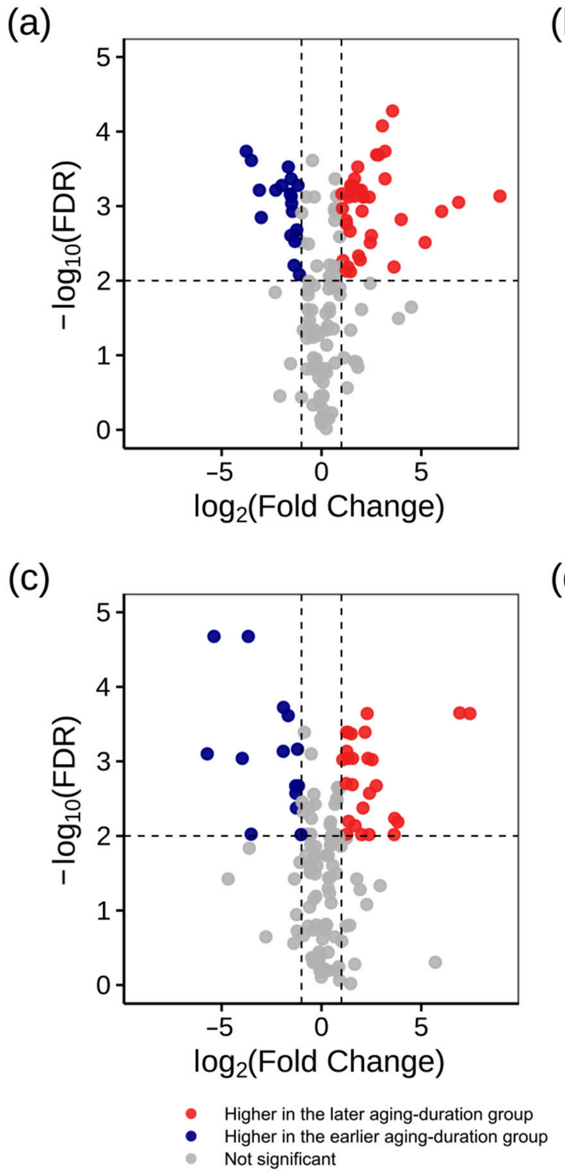

Figure S7. Volcano plots of differential metabolites between AAF-3y and AAF samples stored for 4–7 years. The subplots represent, respectively: (a) AAF-3y vs. AAF-4y, (b) AAF-3y vs. AAF-5y, (c) AAF-3y vs. AAF-6y, and (d) AAF-3y vs. AAF-7y.

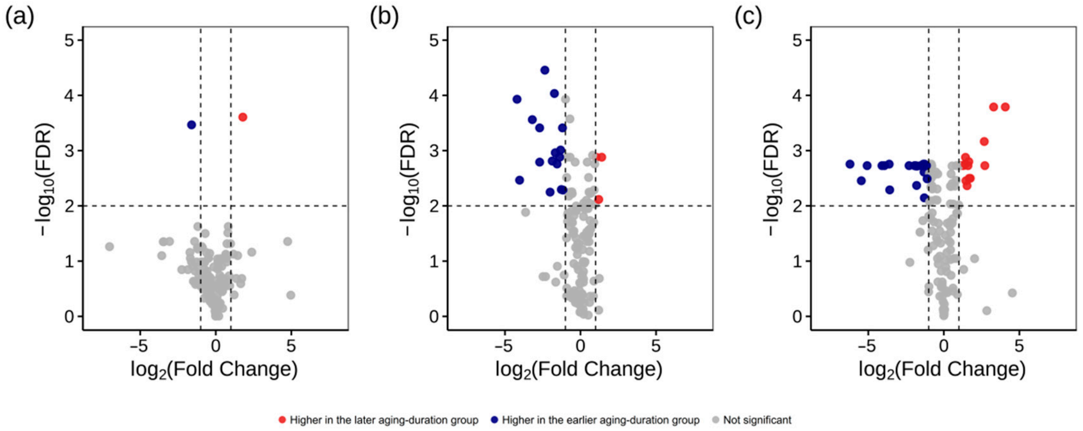

Figure S8. Volcano plots of differential metabolites between AAF-4y and AAF samples stored for 5–7 years. The subplots represent, respectively: (a) AAF-4y vs. AAF-5y, (b) AAF-4y vs. AAF-6y, and (c) AAF-4y vs. AAF-7y.

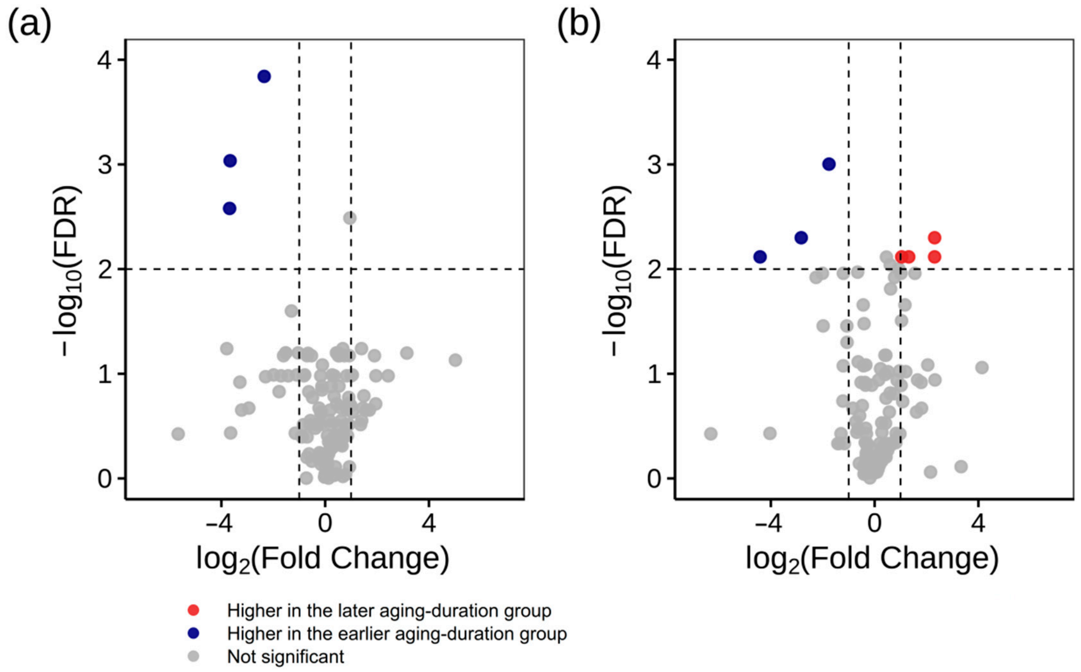

Figure S9. Volcano plots of differential metabolites between AAF-5y and AAF samples stored for 6 and 7 years. The subplots represent, respectively: (a) AAF-5y vs. AAF-6y, (b) AAF-5y vs. AAF-7y.

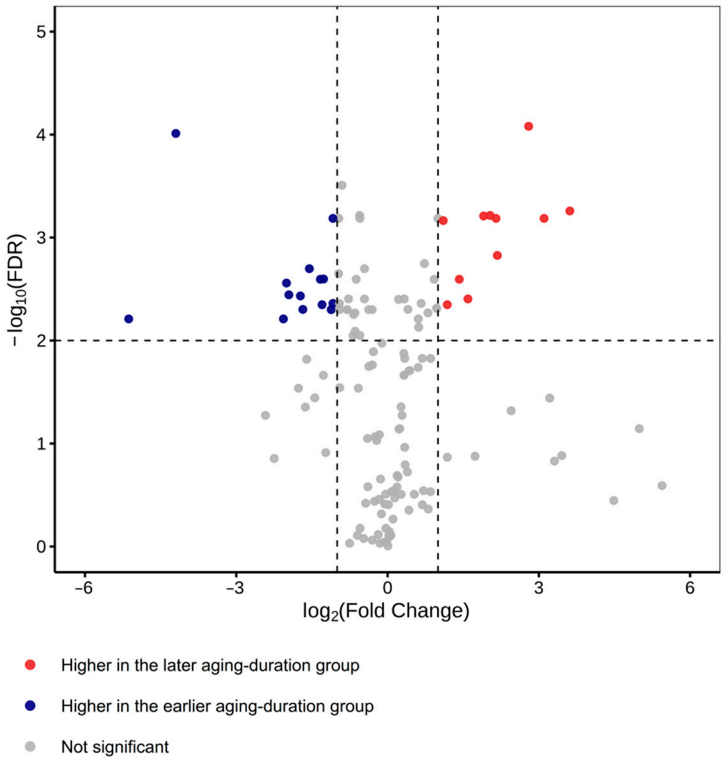

Figure S10. Volcano plot of differential metabolites between AAF-6y and AAF-7y.

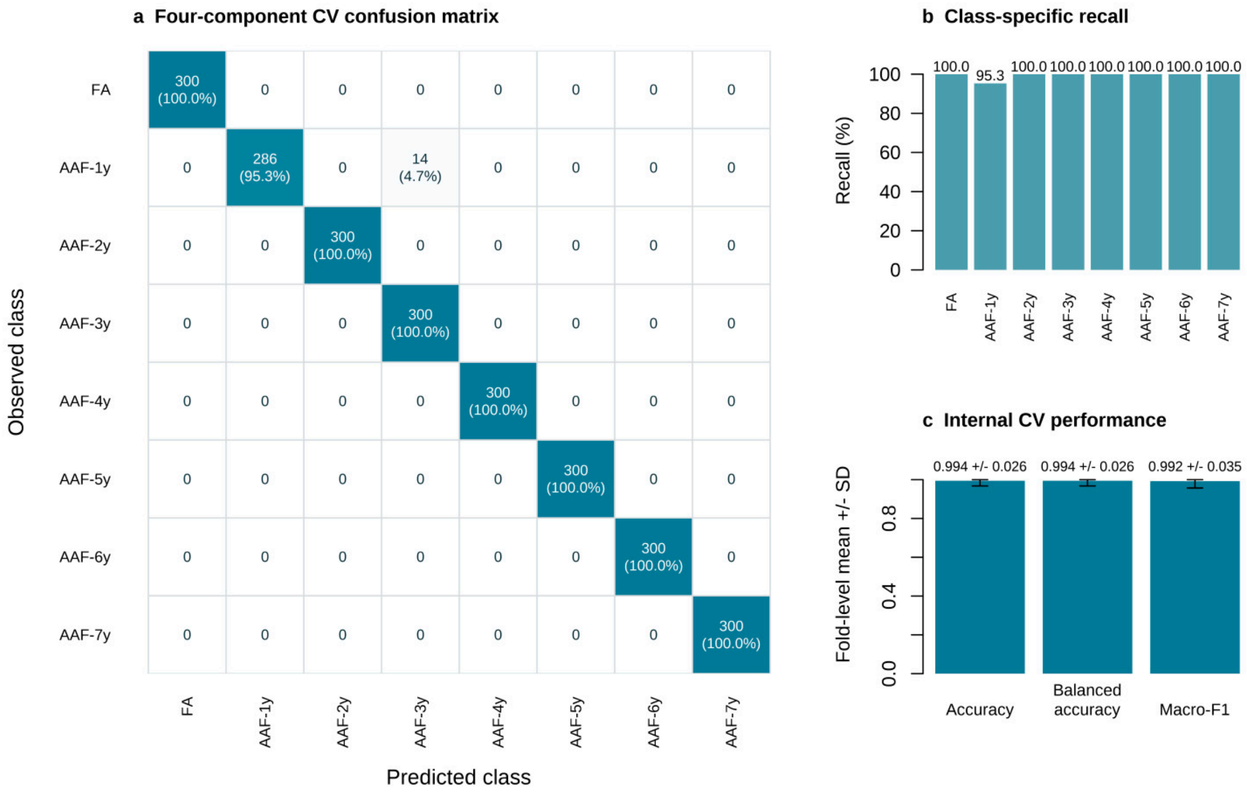

Figure S11. Internal cross-validation of the four-component PLS-DA model. (a) Aggregated out-of-fold prediction confusion matrix, with rows and columns representing true and predicted classes, respectively; percentages in parentheses indicate row percentages; (b) recall rates for each class; (c) mean ± SD of accuracy, balanced accuracy,

and Macro-F1. Results are from 100 repetitions of stratified three-fold cross-validation; error bars represent the standard deviation across 300 test folds, truncated to [0,1]. The 2400 prediction records were derived from duplicate tests of 24 samples.

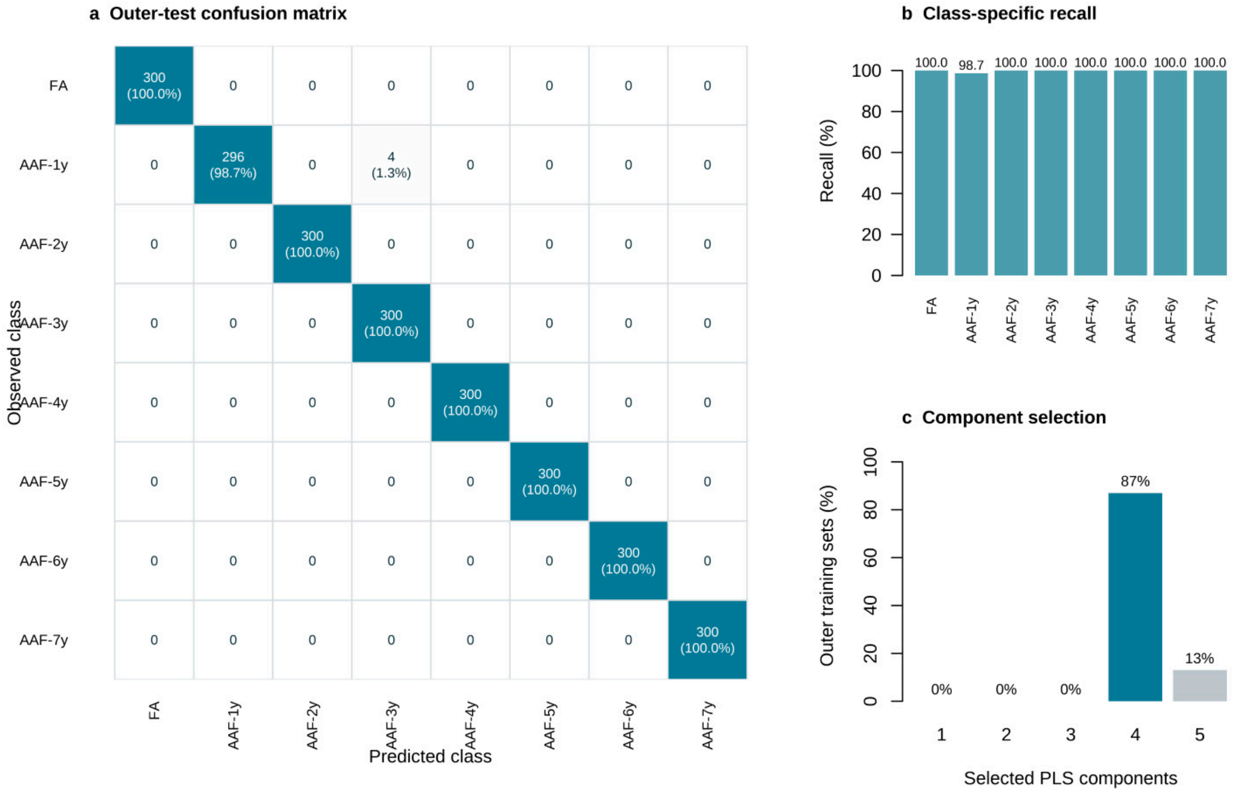

Figure S12. (a) Aggregated confusion matrix of outer-layer predictions, where rows and columns represent true and predicted classes, respectively; percentages in parentheses indicate row percentages; (b) Recall rates for each class; (c) Component selection frequencies. The outer layer employed 100 repetitions of stratified three-fold cross-validation, and within each outer-layer training set, 10 repetitions of stratified two-fold cross-validation were used to select the number of components. For models with 1–5 components, the model with the fewest components and an average inner-layer accuracy differing by no more than 1 percentage point from the maximum value was selected. The 2400 prediction records were derived from replicate tests of 24 biological samples (3 per group), with each sample tested 100 times. These results evaluate the component selection process rather than a fixed four-component model. FA denotes fresh AAF, while AAF-1y–AAF-7y denotes samples aged 1–7 years.

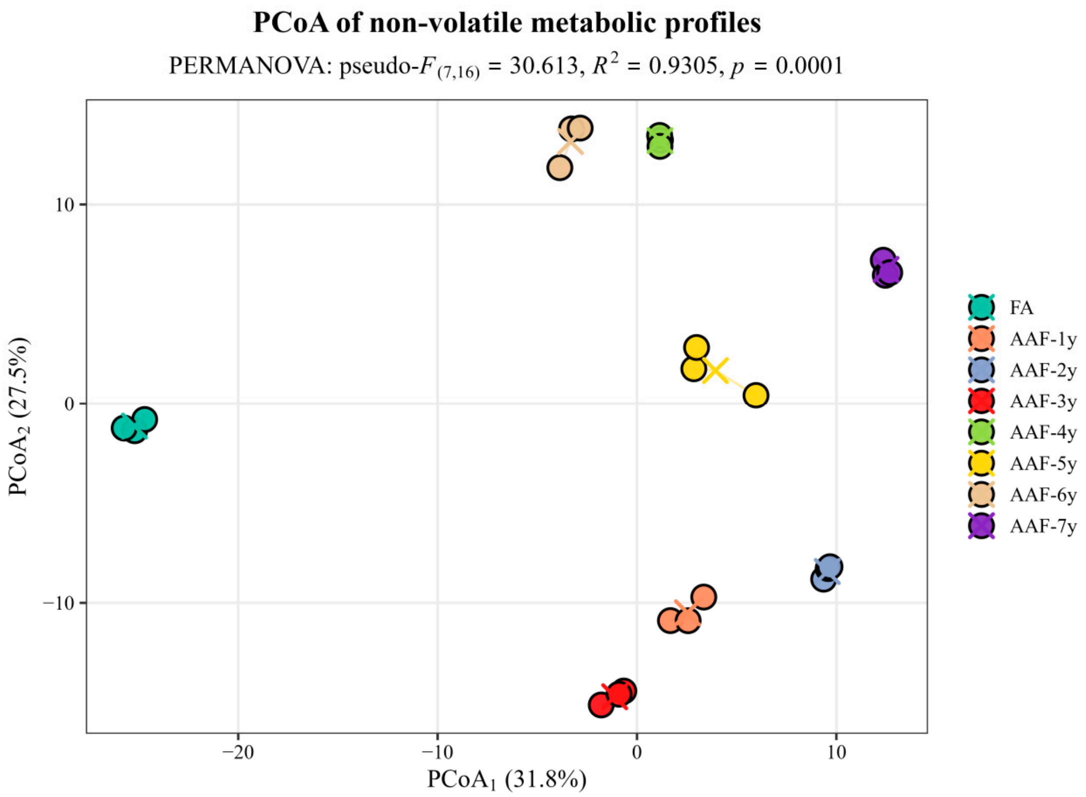

Figure S13. Principal coordinate analysis (PCoA) of the non-volatile metabolic profiles of AAF samples across aging-duration groups. Euclidean distances were calculated from the Pareto-scaled data matrix. Circles represent individual biological samples (n = 3 per group); crosses indicate group centroids, and lines connect samples to their respective centroids. $PCoA_1$ and $PCoA_2$ explain 31.8% and 27.5% of the variation, respectively. Overall group differences were assessed using PERMANOVA with 9999 permutations (F = 30.613, R^2 = 0.9305, p = 0.0001).

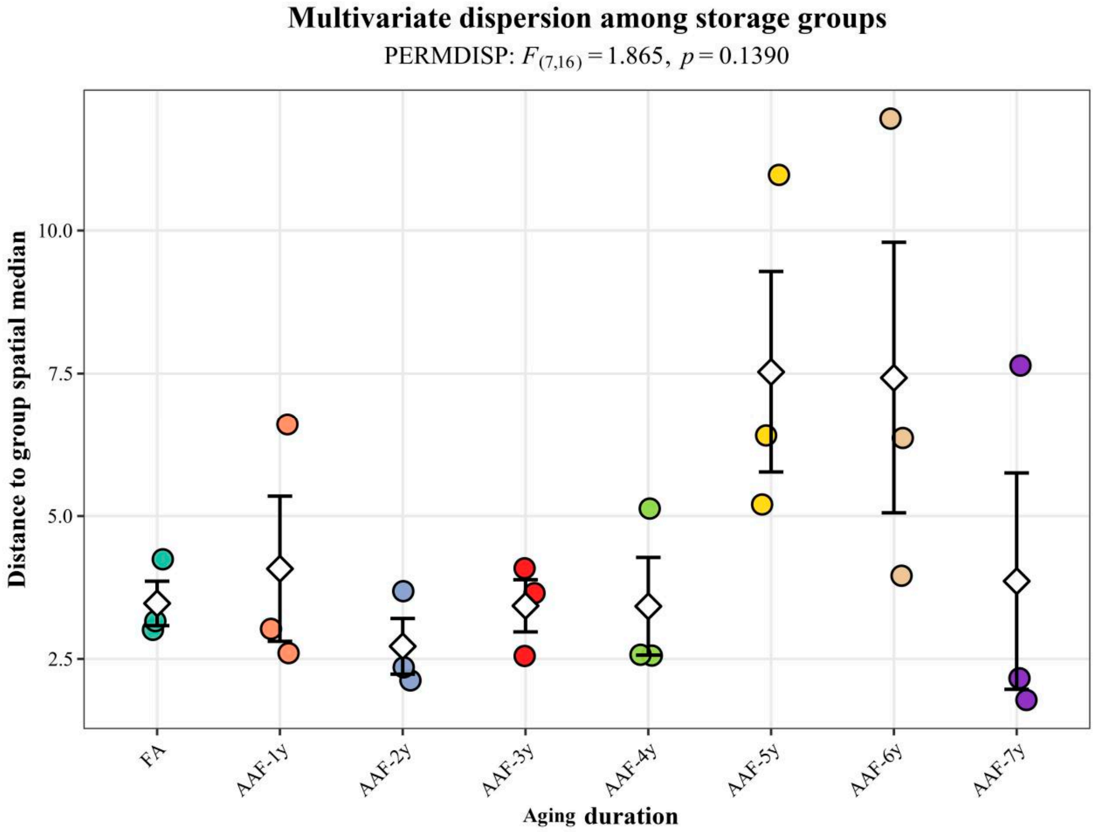

Figure S14. Homogeneity of multivariate dispersions among AAF aging-duration groups. Colored circles represent individual sample distances to the spatial median of their respective groups. Open diamonds and error bars indicate group means ± SE (*n* = 3 per group). Multivariate dispersion was assessed using PERMDISP with 9999 permutations (F = 1.865, *p* = 0.1390).

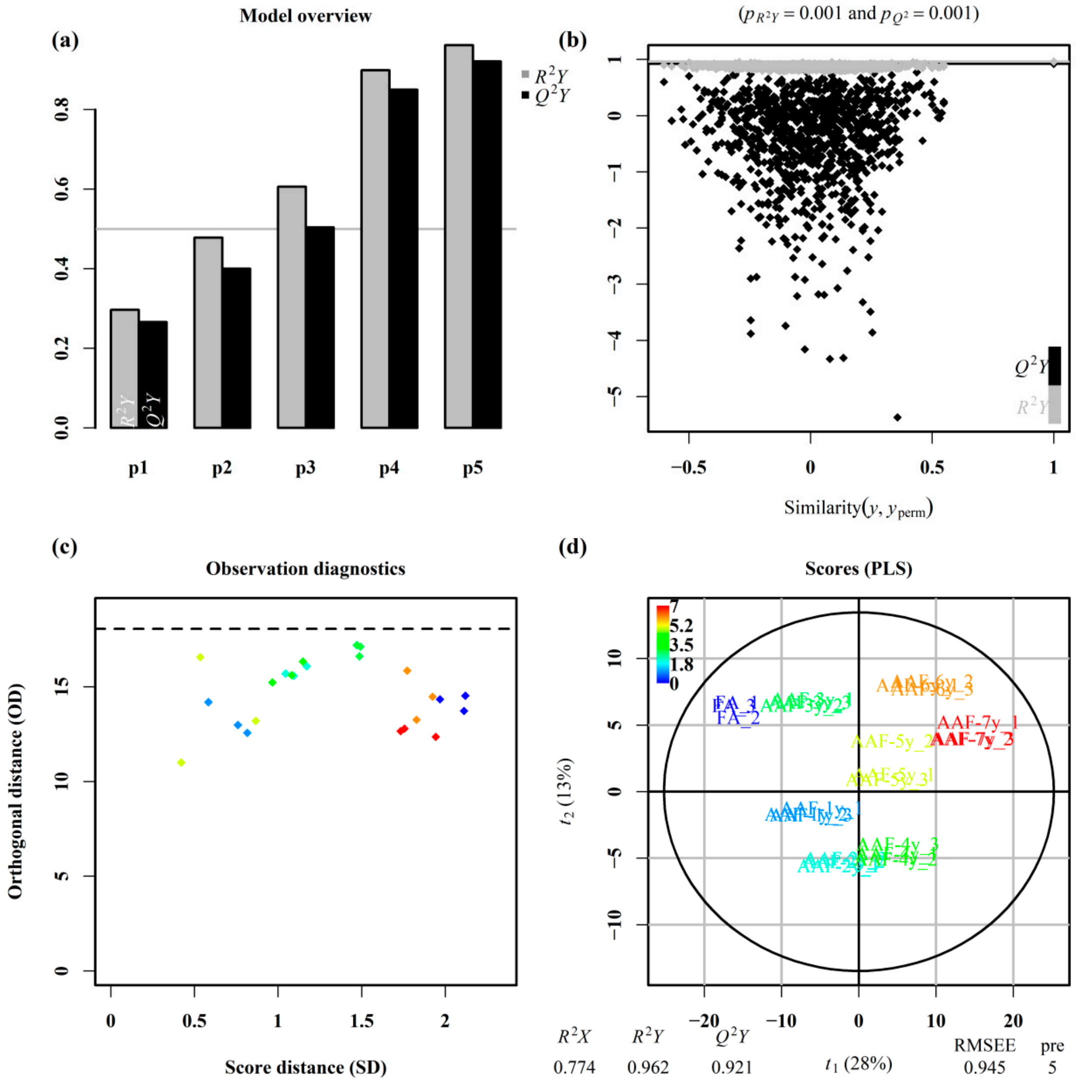

Figure S15. Diagnostic evaluation of the PLS regression model for eight groups of AAF samples with different aging durations. (a) Cumulative R^2Y and cross-validated Q^2 for models with one to five predictive components; gray and black bars represent R^2Y and $Q^{2,}$ respectively; (b) Results of the 1000-permutation test (p = 0.001 for both R^2Y and Q^2); (c) Observation diagnostic plot showing the score distance and orthogonal distance of each sample. The dashed lines indicate the model-derived diagnostic limits used to identify potentially unusual observations; (d) Score plot based on the first two predictive components of the five-component PLS regression model. The model yielded cumulative R^2X = 0.77, R^2Y = 0.96, and Q^2 = 0.92, with a root mean squared error of estimation (RMSEE) of 0.95.

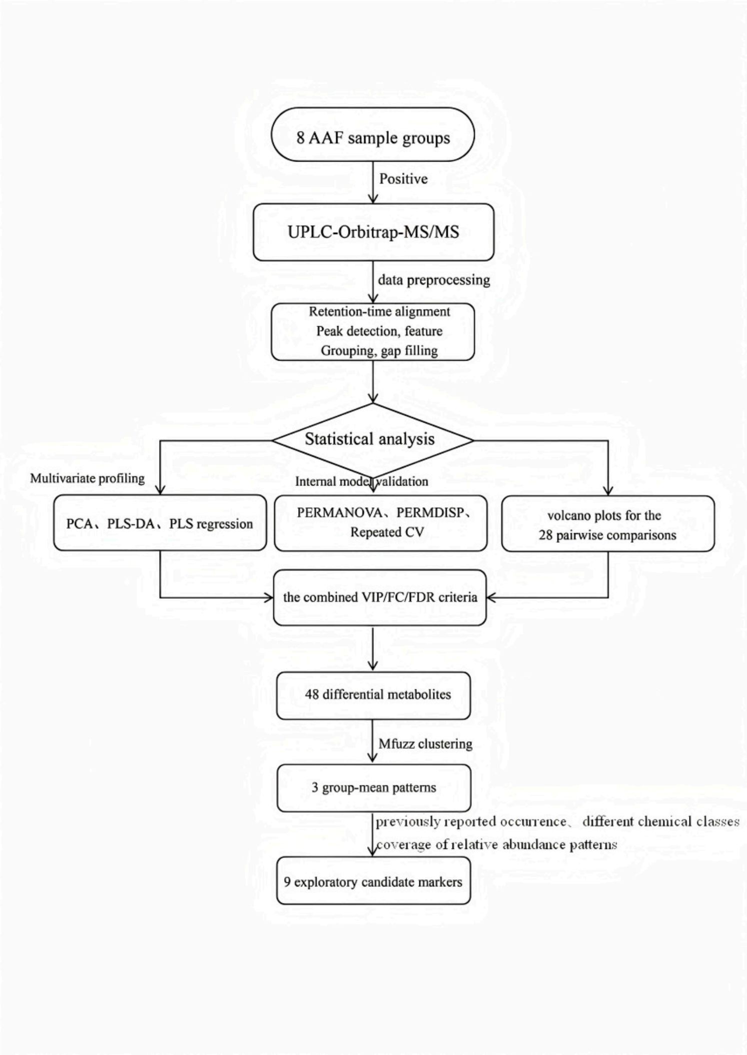

Figure S16. Workflow of UPLC-Orbitrap-MS/MS-based metabolomic analysis and exploratory candidate markers selection in AAF samples with different aging durations.

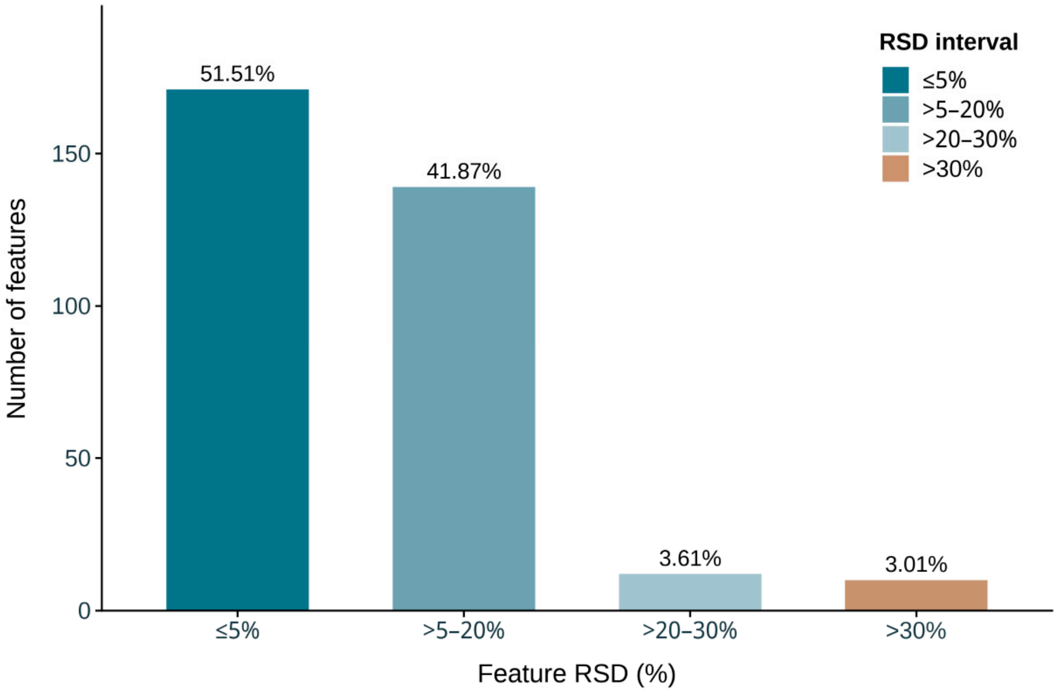

Figure S17. Distribution of feature relative standard deviations (RSDs) values across all QC samples.

Table S1. Putatively annotated non-volatile metabolites in AAF samples representing different aging durations in positive-ion mode.

No.	*t*R (min)	Molecular Formula	Ion mode	Measured*m/z*	Theoretical*m/z*	Mass Error(ppm)	Component name	MS/MS fragments (HCD)	Compound classification	Confidence level
1	1.03	$C_6H_{11}NO_2$	$[M+H]^+$	130.0866	130.0863	2.31	*L*-Pipecolate	84.0807, 70.0651, 56.0495, 112.0753	Amino acids and derivatives	2
2	1.05	$C_7H_{13}NO_2$	$[M+H]^+$	144.1023	144.1019	2.78	Proline betaine	58.0653, 84.0811, 102.0557, 144.1026	Amino acids and derivatives	2
3	1.06	$C_6H_{13}NO_2$	$[M+H]^+$	132.1022	132.1019	2.27	*L*-Isoleucine	69.0698, 89.0964, 132.1021	Amino acids and derivatives	2
4	1.23	$C_5H_{11}NO_2$	$[M+H]^+$	118.0866	118.0863	2.54	*L*-Valine	55.0543, 72.0809, 118.0866	Amino acids and derivatives	2
5	1.28	$C_5H_5N_5$	$[M+H]^+$	136.0621	136.0618	2.20	Adenine	119.0355, 136.0621, 137.0462, 137.0749	Others	2
6	1.38	$C_6H_{11}NO_2$	$[M+H]^+$	130.0866	130.0863	2.31	2-Pyrrolidineacetic acid	84.0812, 130.0870	Amino acids and derivatives	2
7	1.38	$C_5H_{11}NO_2$	$[M+H]^+$	118.0865	118.0863	1.69	5-Aminopentanoate	118.0863, 72.0808, 55.0542	Amino acids and derivatives	2
8	1.43	$C_5H_7NO_3$	$[M+H]^+$	130.0503	130.0499	3.08	*L*-Pyroglutamic Acid	56.0500, 84.0811	Amino acids and derivatives	2
9	1.43	$C_7H_{11}NO_5$	$[M+H]^+$	190.0716	190.0710	3.16	*N*-Acetyl-L-glutamate	84.0445, 102.0551, 130.0499, 148.0608	Amino acids and derivatives	2
10	1.48	$C_9H_{10}O_3$	$[M+H]^+$	167.0708	167.0703	2.99	3-(3-Hydroxyphenyl)propionate	107.0492, 121.0651, 167.0708	Phenolic acids	2
11	1.49	$C_5H_9NO_2$	$[M+H]^+$	116.0708	116.0706	1.72	*D*-Proline	79.0652, 116.0709	Amino acids and derivatives	2
12	1.81	$C_8H_{11}NO$	$[M+H-H_2O]^+$	120.0809	120.0807	1.67	Phenylethanolamine	93.0704, 103.0547, 120.0810	Others	2
13	2.02	$C_9H_{17}NO_5$	$[M+H]^+$	220.1185	220.1179	2.73	Vitamin B5	70.0290, 90.0552, 98.0239, 124.0762	Others	2

14	2.25	$C_8H_{11}NO_3$	$[M+H]^+$	170.0816	170.0812	2.35	Pyridoxine	55.0542, 80.0492, 84.0449, 96.0449, 152.0712	Others	2
15	2.43	$C_7H_7NO_2$	$[M+H]^+$	138.0553	138.0550	2.17	Anthranilate	92.0498, 120.0447, 138.0553	Organic acids	2
16	2.44	$C_7H_8O_4$	$[M+H]^+$	157.0499	157.0495	2.55	(2*S*,3*S*)-2,3-Dihydroxy-2,3-dihydrobenzoate	65.0385, 69.0335, 83.0492, 93.0335, 97.0284, 111.0442, 121.0285, 139.0389, 157.0499	Organic acids	2
17	2.89	$C_{11}H_{12}N_2O_2$	$[M+H]^+$	205.0976	205.0972	1.95	*L*-Tryptophan	67.0181, 85.0285, 107.0491, 121.0286, 147.0444, 153.0186, 171.0290, 179.0344, 189.0546	Amino acids and derivatives	2
18	2.89	$C_{11}H_9NO_2$	$[M+H]^+$	188.0710	188.0706	2.13	3-Indoleacrylic acid	132.0812, 170.0604, 188.0712	Amino acids and derivatives	2
19	2.89	$C_7H_{10}O_4$	$[M+H]^+$	159.0654	159.0652	1.26	2-Isopropylmaleate	95.0492, 113.0599, 138.0549	Organic acids	2
20	3.12	$C_6H_6O_3$	$[M+H]^+$	127.0392	127.0390	1.57	5-Hydroxymethylfurfural	81.0334, 109.0287, 127.0393	Others	2
21	3.20	$C_{15}H_{16}O_9$	$[M+H]^+$	341.0877	341.0867	2.93	Esculin	123.0442, 151.0393, 179.0342	Lignans and coumarins	2
22	3.30	$C_{11}H_9NO_2$	$[M+H]^+$	188.0712	188.0706	3.19	3-amino-2-naphthoic acid	142.0651, 160.0757, 170.0600, 188.0706	Organic acids	2
23	3.64	$C_7H_8O_3$	$[M+H]^+$	141.0548	141.0546	1.49	Gentisyl Alcohol or isomers	138.0551, 126.0315	Others	2
24*	3.68	$C_{21}H_{18}O_{13}$	$[M+H]^+$	479.0833	479.0820	2.71	Querciturone	153.0190, 285.0409, 303.0514	Flavonoids	2
25*	3.70	$C_{15}H_{16}O_8$	$[M+H]^+$	325.0925	325.0918	2.15	Umbelliferone-7-*O*-glucoside or isomers	135.0448, 163.0398	Lignans and coumarins	2
26	3.83	$C_9H_6O_3$	$[M+H]^+$	163.0393	163.0390	1.84	Umbelliferone	77.0388, 89.0388, 145.0292, 163.0396	Lignans and coumarins	2
27	3.84	$C_{16}H_{18}O_9$	$[M+H]^+$	355.1029	355.1024	1.41	Chlorogenic acid or isomers	135.0442, 163.0391	Phenolic acids	2
28	3.91	$C_9H_8O_3$	$[M+H]^+$	165.0550	165.0546	2.42	*p*-Coumaric acid	91.0546, 119.0497, 147.0449, 148.0490	Phenolic acids	2
29	3.94	$C_{10}H_8O_4$	$[M+H]^+$	193.0500	193.0495	2.59	Isoscopoletin or isomers	122.0366, 133.0289, 137.0602, 178.0267	Lignans and coumarins	2
30	4.19	$C_{10}H_8O_4$	$[M+H]^+$	193.0500	193.0495	2.59	Scopoletin or isomers	122.0367, 133.0288, 178.0266	Lignans and	2

									coumarins	
31*	4.24	$C_{20}H_{29}NO_6$	[M+H]+	380.2073	380.2068	1.32	Pulchellamine E or isomers	95.0491, 131.0855, 220.1332, 268.1543, 300.1958, 380.2068	Terpenoids	2
32*	4.42	$C_{19}H_{25}NO_4$	$[M+H]^+$	332.1864	332.1856	2.41	Erythratidine or isomers	70.0652, 82.0654, 84.0809, 96.0444, 110.0966, 154.0866, 314.1743, 286.1797	Others	2
33*	4.43	$C_{17}H_{22}O_6$	$[M+H]^+$	323.1498	323.1489	2.79	Tetraneurin A or isomers	95.0491, 191.1067, 193.1223, 203.1067, 247.1329	Terpenoids	2
34	4.61	$C_{10}H_{11}NO_3$	$[M+H]^+$	194.0816	194.0812	2.06	*N*-Phenylacetylglycine	91.0542, 93.0699, 103.0543, 148.0760, 194.0825	Amino acids and derivatives	2
35	4.61	$C_9H_{11}NO_2$	$[M+H]^+$	166.0867	166.0863	2.41	*L*-Phenylalanine	120.0811, 166.0867, 103.0546	Amino acids and derivatives	2
36	4.66	$C_{26}H_{28}O_{14}$	$[M+H]^+$	565.1567	565.1552	2.65	Schaftoside or isomers	121.0287, 207.0293, 283.0608, 295.0607, 325.0713, 351.0869, 403.0843	Flavonoids	2
37*	4.67	$C_{20}H_{24}O_6$	$[M+NH_4]^+$	378.1919	378.1911	2.12	[(3a*R*,4*S*,6*Z*,9*Z*,11a*S*)-4-Hydroxy-6,10-dimethyl-3-methylidene-2,8-dioxo-4,5,11,11a-tetrahydro-3a*H*-cyclodeca[b]furan-11-yl] (*Z*)-2-methylbut-2-enoate or isomers	147.0801, 155.0849, 119.0847, 129.0683	Terpenoids	2
38	4.75	$C_{20}H_{25}NO_6$	$[M+H]^+$	376.1762	376.1760	0.53	6-*O*-Methyl-N-deacetylisoipecoside Aglycone or isomers	70.0648, 176.1070, 272.1281, 330.1697	Others	2
39*	4.76	$C_{25}H_{24}O_{12}$	$[M+H]^+$	517.1351	517.1341	1.93	Isochlorogenic acid A or isomers	145.0294, 163.0395, 499.1230	Phenolic acids	2
40*	4.86	$C_{15}H_{20}O_3$	$[M+H]^+$	249.1491	249.1485	2.41	2α-Hydroxyalantolactone or isomers	231.1375, 213.1269, 185.1321	Terpenoids	2
41*	4.91	$C_{13}H_{14}O_2$	$[M+H]^+$	203.1072	203.1067	2.46	Tremetone or isomers	123.0441, 133.0282, 151.0388, 179.0336	Others	2
42*	4.92	$C_9H_6O_4$	$[M+H]^+$	179.0343	179.0339	2.23	Esculetin	179.0344, 123.0444, 133.0288, 135.0445, 151.0394	Lignans and coumarins	2
43*	5.07	$C_9H_6O_2$	$[M+H]^+$	147.0443	147.0441	1.36	Coumarin	91.0543, 95.0492, 103.0543, 119.0494, 147.0443	Lignans and coumarins	2

44	5.14	$C_{10}H_8O_3$	$[M+H]^+$	177.0551	177.0546	2.82	7-Methoxycoumarin	118.0406, 121.0695, 133.0646, 177.0543	Lignans and coumarins	2
45	5.14	$C_{17}H_{20}O_9$	$[M+H]^+$	369.1189	369.1180	2.44	5-Feruloylquinic acid	145.0291, 163.0397, 177.0555	Phenolic acids	2
46	5.23	$C_{27}H_{30}O_{16}$	$[M+H]^+$	611.1623	611.1607	2.62	Luteolin-7-*O*-gentiobioside	269.0467, 287.0565	Flavonoids	2
47	5.29	$C_{21}H_{20}O_{11}$	$[M+H]^+$	449.1087	449.1078	2.00	Kaempferol 7-*O*-glucoside	287.0562, 449.1078	Flavonoids	2
48	5.41	$C_{20}H_{22}O_4$	$[M+H]^+$	327.1599	327.1591	2.45	Dehydrodiisoeugenol	103.0547, 137.0605, 151.0762, 187.0766, 235.1127	Lignans and coumarins	2
49	5.56	$C_{15}H_{18}O_5$	$[M+H]^+$	279.1232	279.1227	1.79	Tanaphillin or isomers	95.0491, 107.0491, 111.0441, 121.0648, 123.0804, 137.0597, 177.0546, 203.0703, 215.1016, 219.1016, 233.1172	Terpenoids	2
50*	5.64	$C_{15}H_{18}O_3$	$[M+H]^+$	247.1333	247.1329	1.62	Santonin	153.0184, 241.0496, 287.0551	Terpenoids	2
51*	5.64	$C_{27}H_{30}O_{16}$	$[M+H]^+$	611.1625	611.1607	2.95	Rutin	85.0285, 303.0507	Flavonoids	2
52	5.74	$C_{21}H_{20}O_{12}$	$[M+H]^+$	465.1041	465.1028	2.80	Hyperoside	153.0188, 285.0404, 303.0507	Flavonoids	2
53	5.76	$C_{21}H_{20}O_{10}$	$[M+H]^+$	433.1140	433.1129	2.54	Vitexin or isomers	165.0189, 283.0611, 313.0717, 349.0729, 379.0837	Flavonoids	2
54	5.78	$C_{21}H_{28}O_9$	$[M+H]^+$	425.1816	425.1806	2.35	Grandidentatin or isomers	69.0336, 73.0284, 85.0286, 97.0287, 105.0702, 147.0440, 161.0590, 163.0760, 263.1300	Others	2
55	5.83	$C_{27}H_{30}O_{15}$	$[M+H]^+$	595.1676	595.1657	3.19	Biorobin	153.0187, 269.0452, 287.0558	Flavonoids	2
56	5.93	$C_{21}H_{20}O_{12}$	$[M+H]^+$	465.1041	465.1028	2.80	Isoquercitrin	85.0283, 153.0186, 229.0502, 303.0505	Flavonoids	2
57	5.97	$C_{21}H_{20}O_{11}$	$[M+H]^+$	449.1091	449.1078	2.89	Kaempferol-*O*-hexoside	287.0558, 449.1078	Flavonoids	2
58*	6.00	$C_{22}H_{22}O_{11}$	$[M+H]^+$	463.1231	463.1235	-0.86	Diosmetin-7-*O*-Galactoside	301.0704, 286.0467, 463.1248	Flavonoids	2
59	6.00	$C_{21}H_{18}O_{12}$	$[M+H]^+$	463.0879	463.0877	0.43	Luteolin 7-Glucuronide	153.0187, 287.0555	Flavonoids	2
60	6.29	$C_{27}H_{30}O_{14}$	$[M+H]^+$	579.1721	579.1708	2.24	Rhoifolin	271.0605, 433.1127	Flavonoids	2
61*	6.37	$C_{11}H_{10}O_5$	$[M+H]^+$	223.0604	223.0601	1.35	Isofraxidin	162.0317, 190.0267, 207.0295, 223.0608	Lignans and coumarins	2
62*	6.48	$C_{15}H_{10}O_6$	$[M+H]^+$	287.0557	287.0550	2.44	Luteolin	137.0240, 151.0190, 213.0557, 258.0535, 287.0563	Flavonoids	2
63*	6.52	$C_{15}H_{18}O_3$	$[M+H]^+$	247.1334	247.1329	2.02	Arglabin or isomers	131.0859, 159.1173, 201.1280, 229.1225, 247.1334	Terpenoids	2

64*	6.57	$C_{28}H_{32}O_{15}$	$[M+H]^+$	609.1835	609.1814	3.45	Diosmin or isomers	151.0185, 301.0716	Flavonoids	2
65	6.59	$C_{15}H_{12}O_6$	$[M+H]^+$	289.0714	289.0707	2.42	(+)-Dihydrokaempferol	153.0181, 271.0598, 289.0701	Flavonoids	2
66*	6.61	$C_{22}H_{22}O_{12}$	$[M+H]^+$	479.1197	479.1184	2.71	Nepitrin	245.0453, 301.0351, 317.0668	Flavonoids	2
67	6.72	$C_{21}H_{18}O_{11}$	$[M+H]^+$	447.0934	447.0922	2.68	Apigenin 7-*O*-Glucuronide or isomers	119.0496, 153.0190, 271.0613	Flavonoids	2
68	6.78	$C_{11}H_{16}O_3$	$[M+H]^+$	197.1177	197.1172	2.54	Loliolide	133.1018, 161.0969, 179.1076	Terpenoids	2
69	6.80	$C_{21}H_{20}O_{11}$	$[M+H]^+$	449.1091	449.1083	1.78	Cyanidin 5-O-glucoside	153.0191, 271.0614, 273.0773	Flavonoids	2
70	6.84	$C_{22}H_{22}O_{11}$	$[M+H]^+$	463.1247	463.1235	2.59	Homoplantaginin	229.0487, 285.0399, 301.0719	Flavonoids	2
71	6.84	$C_{16}H_{12}O_6$	$[M+H]^+$	301.0714	301.0707	2.33	Diosmetin	286.0485, 301.0721	Flavonoids	2
72	6.89	$C_{22}H_{20}O_{12}$	$[M+H]^+$	477.1040	477.1028	2.52	Diosmetin-7-*O*-Glucuronide	286.0485, 301.0721	Flavonoids	2
73	7.07	$C_{15}H_{20}O_3$	$[M+H]^+$	249.1491	249.1490	0.40	Parthenolide or isomers	81.0700, 123.0808, 131.1015, 171.1175, 203.1437, 231.1384	Terpenoids	2
74	7.15	$C_{10}H_{10}O_3$	$[M+H]^+$	179.0707	179.0703	2.23	Ferulaldehyde	91.0545, 119.0496, 147.0447, 179.0349	Others	2
75	7.25	$C_{15}H_{20}O_4$	$[M+H]^+$	265.1439	265.1434	1.89	Abscisic acid	119.0862, 159.1179, 187.1128, 211.1129	Terpenoids	2
76*	7.88	$C_{16}H_{20}O_5$	$[M+H]^+$	293.1391	293.1384	2.39	11,12-Dimethoxydihydrokawain or isomers	109.0649, 151.0760, 177.0912, 215.1073	Others	2
77	7.91	$C_{24}H_{26}O_{12}$	$[M+H]^+$	507.1511	507.1497	2.76	Eupatilin-*O*-Hexoside or isomers	315.0509, 345.0984	Flavonoids	2
78	7.97	$C_{15}H_{10}O_7$	$[M+H]^+$	303.0506	303.0499	2.31	Quercetin	247.0594, 257.0444, 285.0392, 275.0538	Flavonoids	2
79*	8.02	$C_{20}H_{28}O_6$	$[M+H]^+$	365.1963	365.1959	1.10	Argyinolide H	57.0699, 109.0284, 227.1067, 245.1172,263.1278, 281.1384, 323.1489, 347.1853, 365.1959	Terpenoids	2
80	8.08	$C_{25}H_{24}O_{12}$	$[M+H]^+$	517.1353	517.1341	2.32	Cynarine	163.0387, 499.1220, 337.0922	Phenolic acids	2
81	8.20	$C_{16}H_{12}O_8$	$[M+H]^+$	333.0612	333.0605	2.10	Laricitrin	219.0304,318.0387,333.0624	Flavonoids	2
82	8.23	$C_{15}H_{12}O_6$	$[M+H]^+$	289.0711	289.0707	1.38	Eriodictyol	153.0190, 163.0398, 179.0344, 253.0514	Flavonoids	2
83*	8.38	$C_{15}H_{16}O_2$	$[M+H]^+$	229.1228	229.1223	2.18	Mansonone C	93.0699, 119.0853, 145.1007, 157.1012, 183.1168, 201.1274, 211.1117	Terpenoids	2
84	8.38	$C_{17}H_{22}O_5$	$[M+H]^+$	307.1541	307.1540	0.33	(9a-Hydroxy-3,8a-dimethyl-5-methylidene-2-oxo-4,4a,6,7,8,9-hexahydrobenzo[f][1]benzofuran-8-yl) Acetate or isomers	81.0702, 107.0498, 175.1123, 183.1176, 201.1282, 203.1079, 229.1231, 247.1327	Terpenoids	2

85*	8.47	$C_{15}H_{18}O_2$	$[M+H]^+$	231.1384	231.1380	1.73	Dehydrocostus lactone	131.0858, 143.0859, 185.1329, 231.1387	Terpenoids	2
86	8.49	$C_{16}H_{12}O_7$	$[M+H]^+$	317.0660	317.0656	1.26	Isorhamnetin	229.0493, 153.0184, 302.0429	Flavonoids	2
87*	8.67	$C_{23}H_{24}O_{12}$	$[M+H]^+$	493.1368	493.1346	4.46	Tricin-7-*O*-Glucoside	315.0524, 331.0827	Flavonoids	2
88	8.87	$C_{15}H_{24}O$	$[M+H]^+$	221.1905	221.19	2.26	Caryophyllene Oxide or isomers	165.1276, 203.1795, 221.1901	Terpenoids	2
89*	9.02	$C_{10}H_{10}O_4$	$[M+H-H_2O]^+$	177.0551	177.0546	2.82	Ferulic acid	117.0332, 134.0371, 145.0283, 177.0545	Phenolic acids	2
90*	9.21	$C_{15}H_{12}O_5$	$[M+H]^+$	273.0765	273.0758	2.56	Naringenin chalcone	123.0447, 147.0450, 153.0191, 273.0757	Flavonoids	2
91	9.36	$C_{15}H_{12}O_5$	$[M+H]^+$	273.0760	273.0758	0.73	Naringenin	119.0493, 189.1649, 203.1803, 145.0218, 153.0186	Flavonoids	2
92*	9.48	$C_{15}H_{26}O_2$	$[M+H]^+$	239.2010	239.2006	1.67	Bisabolol oxide B or isomers	95.0860, 121.1018, 203.1807	Terpenoids	2
93*	9.50	$C_{15}H_{24}O$	$[M+H]^+$	221.1906	221.1900	2.71	Farnesal	95.0858, 109.1017, 133.1017, 203.1803, 221.1910	Terpenoids	2
94*	9.56	$C_{15}H_{10}O_6$	$[M+H]^+$	287.0554	287.055	1.39	Kaempferol	165.0190, 213.0558, 287.0559, 258.0525	Flavonoids	2
95	9.58	$C_{16}H_{14}O_6$	$[M+H]^+$	303.0874	303.0863	3.63	Hesperetin	177.0554, 153.0189, 285.0515	Flavonoids	2
96*	9.75	$C_{16}H_{12}O_7$	$[M+H]^+$	317.0659	317.0656	0.95	Eupafolin	168.0056, 186.0161, 302.0426, 317.0659	Flavonoids	2
97	10.00	C_7H_6O	$[M+H]^+$	107.0494	107.0491	2.80	Benzaldehyde	91.0545, 107.0495	Others	2
98*	10.06	$C_{15}H_{18}O_3$	$[M+H]^+$	247.1335	247.1329	2.43	Tourneforin or isomers	115.0537, 129.0703, 105.0701, 154.0781, 183.1174	Terpenoids	2
99	10.11	$C_{12}H_{18}O_3$	$[M+H]^+$	211.1333	211.1329	1.89	Jasmonic acid	95.0490, 105.0703, 107.0858, 109.0650, 123.0810	Lipids	2
100*	10.32	$C_{19}H_{22}O_7$	$[M+H]^+$	363.1446	363.1438	2.2	Deacetylorientalide or isomers	345.1363, 363.1465	Terpenoids	2
101	10.48	$C_{18}H_{16}O_8$	$[M+H]^+$	361.0923	361.0918	1.38	5,7,3'-Trihydroxy-6,4',5'-trimeth oxyflavone	361.0949, 303.1600, 250.1808	Flavonoids	2
102*	10.61	$C_{17}H_{20}O_5$	$[M+H]^+$	305.1399	305.1384	4.92	Matricarin	69.0327, 245.1165, 217.1208, 305.1384	Terpenoids	2
103*	10.69	$C_{30}H_{46}O$	$[M+H]^+$	423.3628	423.3621	1.65	Glochidone or isomers	81.0699, 109.1012, 121.1012, 137.0961, 191.1794, 219.1743, 233.190, 245.2264, 301.2526, 381.3152, 395.367, 423.3621	Terpenoids	2
104*	10.7	$C_{15}H_{22}O$	$[M+H]^+$	219.1749	219.1743	2.74	Germacrone or isomers	111.0810, 204.1519, 219.1754	Terpenoids	2
105	10.93	$C_{16}H_{18}O_6$	$[M+H]^+$	307.1182	307.1176	1.95	Cimifugin or isomers	107.049, 120.0599, 148.0521, 163.0391, 176.0470, 203.0339, 221.0446	Others	2
106	10.99	$C_{18}H_{30}O_3$	$[M+H]^+$	295.2274	295.2268	2.03	Colneleate	81.0701, 95.0860, 163.1122, 277.2166	Lipids	2

107	11.07	$C_{17}H_{14}O_6$	$[M+H]^+$	315.0868	315.0863	1.59	Cirsimaritin	272.0634, 300.0628, 315.0872	Flavonoids	2
108	11.28	$C_{19}H_{18}O_8$	$[M+H]^+$	375.1083	375.1074	2.40	Casticin	243.0661, 331.0818, 375.1091	Flavonoids	2
109	11.38	$C_{18}H_{16}O_7$	$[M+H]^+$	345.0970	345.0969	0.29	Eupatilin	169.0137, 284.0686, 330.0741	Flavonoids	2
110	11.46	$C_{18}H_{16}O_8$	$[M+H]^+$	361.0915	361.0918	-0.83	Centaureidin	303.0508, 346.0694, 361.0945	Flavonoids	2
111*	11.54	$C_{42}H_{68}O_{14}$	$[M+H]^+$	797.4710	797.4682	3.51	Hederoside F or Isomers	189.1649, 203.1809	Terpenoids	2
112	11.66	$C_{15}H_{20}O_2$	$[M+H]^+$	233.1541	233.1536	2.14	Atractylenolide II	95.0856, 119.0858, 131.0858, 145.1013, 187.1486, 215.1435	Terpenoids	2
113*	11.90	$C_{15}H_{10}O_4$	$[M+H]^+$	255.0656	255.0652	1.57	Chrysin	103.0544, 129.0338, 147.0447, 181.0663, 209.0607, 213.0549, 231.0659, 255.06580	Flavonoids	2
114	12.02	$C_{18}H_{39}NO_3$	$[M+H]^+$	318.3010	318.3003	2.20	4-Hydroxysphinganine	60.0445, 282.2801	Lipids	2
115	12.08	$C_{15}H_{18}O_2$	$[M+H]^+$	231.1384	231.1380	1.73	Atractylenolide I	203.1441, 231.1285, 213.1389, 185.1336	Terpenoids	2
116*	12.09	$C_{15}H_{20}O_3$	$[M+H]^+$	249.1490	249.1485	2.01	Reynosin or isomers	231.1370	Terpenoids	2
117	12.11	$C_{16}H_{12}O_6$	$[M+H]^+$	301.0712	301.0707	1.66	(3*R*)-Sophorol	125.0602, 135.0446, 259.0606, 283.0610, 301.0711	Flavonoids	2
118*	12.21	$C_{15}H_{10}O_5$	$[M+H]^+$	271.0606	271.0601	1.84	Apigenin	153.0187, 271.0607	Flavonoids	2
119*	12.21	$C_{15}H_{22}O_2$	$[M+H]^+$	235.1697	235.1693	1.70	Curcumenol	107.0857, 119.085, 175.1486, 217.1591	Terpenoids	2
120*	12.41	$C_{17}H_{14}O_7$	$[M+H]^+$	331.0827	331.0812	4.53	Jaceosidin	168.0058, 245.0447, 273.0376, 301.0357	Flavonoids	2
121	12.50	$C_{15}H_{22}O_2$	$[M+H]^+$	235.1697	235.1693	1.70	Artemisinic acid	161.1330, 189.1645, 217.1595	Terpenoids	2
122*	12.65	$C_{15}H_{22}O$	$[M+H]^+$	219.1747	219.1743	1.83	α-Cyperone	97.0654, 111.0810, 133.1018, 159.1170, 201.1648, 219.1754	Terpenoids	2
123*	12.72	$C_{16}H_{12}O_5$	$[M+H]^+$	285.0762	285.0757	1.75	Genkwanin	271.0569, 242.0584, 229.0491, 285.0768	Flavonoids	2
124*	12.78	$C_{25}H_{34}O_5$	$[M+H]^+$	415.2489	415.2479	2.41	8,10-Dihydroxy-3-longipinen-5-one, Diangeloyl or isomers	83.0476, 233.1538, 215.1442, 145.1021, 149.0973	Terpenoids	2
125	12.93	$C_{19}H_{18}O_7$	$[M+H]^+$	359.1132	359.1125	1.95	Santaflavone	163.0760, 271.0914, 298.0843, 343.0821, 359.1146	Flavonoids	2
126	12.98	$C_{19}H_{18}O_8$	$[M+H]^+$	375.1082	375.1074	2.13	Chrysosplenetin B	271.0610, 287.0557, 317.0664, 359.0773	Flavonoids	2
127	13.13	$C_{15}H_{20}O_2$	$[M+H]^+$	233.1540	233.1536	1.72	Isoalantolactone	105.0704, 81.0702, 131.0862, 187.1491, 233.1533	Terpenoids	2
128*	13.25	$C_{34}H_{42}O_{12}$	$[M+H]^+$	643.2776	643.2749	4.20	Xylogranatin A or isomers	449.1959, 453.1908, 523.2326, 565.2432,	Terpenoids	2

								625.264, 643.2749		
129	13.56	$C_{20}H_{20}O_8$	$[M+H]^+$	389.1236	389.1231	1.28	Artemetin	345.0966, 243.0663, 331.0828, 303.0886	Flavonoids	2
130	13.70	$C_{16}H_{26}O_2$	$[M+H]^+$	251.2011	251.2006	1.99	Sclareolide	121.1015, 133.1010, 191.1057, 233.1902	Terpenoids	2
131	14.01	$C_{20}H_{22}O_5$	$[M+H]^+$	343.1546	343.1540	1.75	Moxartenolide	69.0335, 81.0335, 105.0699, 109.0284, 119.0855, 131.0855, 147.0804, 171.0804, 175.0754, 197.0961, 215.1067, 243.1016	Terpenoids	2
132*	14.60	$C_{16}H_{24}O_2$	$[M+H]^+$	249.1855	249.1849	2.41	Methyl isocostate or isomers	69.0699, 81.0699, 109.1012, 123.1168, 135.1168, 163.1481, 179.1067, 193.1223, 217.1587, 249.1849	Terpenoids	2
133*	15.02	$C_{16}H_{12}O_4$	$[M+H]^+$	269.0813	269.0808	1.86	Formononetin	269.0818, 254.0584, 213.0000, 237.0547	Flavonoids	2
134	15.84	$C_{18}H_{30}O_2$	$[M+H]^+$	279.2323	279.2319	1.43	α-Linolenate or isomers	81.0700, 95.0857, 109.1015	Lipids	2
135	15.91	$C_{35}H_{42}O_8$	$[M+H]^+$	591.2967	591.2952	2.54	Arteminolide A	81.0335, 105.0699, 119.0855, 121.0648, 131.0855, 133.0648, 147.0804, 171.0804, 173.0961, 189.0910, 229.1223, 455.2217, 471.2166, 473.2323, 481.2585, 489.2272	Terpenoids	2
136*	16.18	$C_{18}H_{32}O_2$	$[M+H]^+$	281.2481	281.2475	2.13	Linoleate	83.0857, 95.0858, 109.1016, 121.1016, 133.1017, 161.1329, 147.1170, 175.1490	Lipids	2
137	16.56	$C_{18}H_{30}O_3$	$[M+H]^+$	295.2273	295.2268	1.69	9-Oxo-10,12-octadecadienoic acid	81.0698, 95.0491, 277.2159, 295.2269	Lipids	2
138	19.85	$C_{30}H_{48}O_3$	$[M+H]^+$	457.3690	457.3676	3.06	Ursolic acid	81.0698, 123.1168, 175.1488, 203.1802	Terpenoids	2

Notes: Numbered according to retention time (tR). MSI level: Confidence levels as defined by the Metabolomics Standards Initiative: Level 2, putatively annotated based on spectral similarity and diagnostic fragments. An asterisk (*) indicates a differential metabolite retained for downstream analysis.

Table S2. Compound Discoverer workflow parameter settings.

Workflow		Untargeted Metabolomics with statistics Detect Unknowns with ID using Online Databases and mzLogic	
Select Spectra	Spectrum Properties Filter	Lower RT Limit	0
		Upper RT Limit	20
	Scan Event Filters	Polarity Mode	Is +
Detect Compounds	General Settings	Mass Tolerance [ppm]	5 ppm
		Min. Peak Intensity	40000
		Use Most Intense Isotope Only	True
	Peak Detection	Chromatographic S/N Threshold	1.5
		Remove Baseline	False
	Isotope Pattern Detection	Group Isotopes for	Br; Cl
	Compound Detection	Ions	[M+H]+1
Group Compounds	General Settings	Mass Tolerance	5 ppm
		RT Tolerance [min]	0.2
		Preferred Ions	[M-H]-1; [M+H]+1
		Area Integration	Most common ion
	Peak Rating Contributions	Area Contribution	3
		CV Contribution	10
		FWHM to Base Contribution	5
		Jaggedness Contribution	5
		Modality Contribution	5
		Zig-Zag Index Contribution	5
	Peak Rating Filter	Peak Rating Threshold	4
		Number of Files	2
Fill Gaps	General Settings	Mass Tolerance	5 ppm
		S/N Threshold	1.5
Predict Compositions	Prediction Settings	Mass Tolerance	5 ppm
		Min. Element Counts	C H
		Max. Element Counts	C90 H190 Br3 Cl4 N10 O18 P3 S5
		Min. RDBE	0
		Max. RDBE	40
		Min. H/C	0.1

		Max. H/C	3.5
		Max. # Candidates	10
	Pattern Matching	Intensity Tolerance [%]	30
		Intensity Threshold [%]	0.1
		S/N Threshold	3
		Use Dynamic Recalibration	True
	Fragments Matching	Use Fragments Matching	True
		Mass Tolerance	5 ppm
		S/N Threshold	3
Mark Background Compounds	General Settings	Max. Sample/Blank	5
		Max. Blank/Sample	0
		Hide Background	True

Table S3. UPLC–Orbitrap MS/MS acquisition parameters recorded in raw data files.

Category	Parameter	Setting
LC conditions	Solvent channel A label	0.1%FA (as recorded in the method)
	Solvent channel B label	ACN (as recorded in the method)
	Flow rate	0.25 mL/min
	Column temperature	40 °C
MS conditions	Mass spectrometer	Orbitrap Elite (Thermo Fisher Scientific)
	Ion source and polarity	Heated electrospray ionization (HESI), positive-ion mode
	Spray voltage	3.50 kV
	Source heater temperature	300 °C
	Capillary temperature	275 °C
	Sheath gas flow	35 arbitrary units
	Auxiliary gas flow	10 arbitrary units
	S-lens RF level	60%
	MS acquisition duration	25 min
	MS1 acquisition	Full scan, m/z 100–2000
	MS1 resolving-power setting	60,000
	MS2 resolving-power setting	15,000
	MS1 AGC target	1×10^6
	MS2 AGC target	5×10^4
	MS1 maximum injection time	200 ms
	MS2 maximum injection time	100 ms
	Microscans	1 for MS1 and MS2
	Data format	Centroid
MS/MS acquisition	Acquisition strategy	Data-dependent MS/MS of the most intense precursor from the preceding MS1 scan (Top-1)

Table S3. UPLC–Orbitrap MS/MS acquisition parameters recorded in raw data files.

Category	Parameter	Setting
	Fragmentation	Higher-energy collisional dissociation (HCD)
	Collision energy (eV)	60
	Isolation width (Da)	2.0
	Minimum precursor signal	500 (instrument setting)
	Default charge state	1
	MS2 first mass	Fixed at m/z 50
	Exclusion mass window	Lower and upper widths, 1.50 each (method settings)
	Precursor selection options	Charge-state screening and monoisotopic precursor selection disabled
File metadata	Instrument software	2.7.0 SP2
